# Projected Scenarios for Coastal First Nations’ Fisheries Catch Potential under Climate Change: Management Challenges and Opportunities

**DOI:** 10.1371/journal.pone.0145285

**Published:** 2016-01-13

**Authors:** Lauren V. Weatherdon, Yoshitaka Ota, Miranda C. Jones, David A. Close, William W. L. Cheung

**Affiliations:** 1 Changing Ocean Research Unit, Global Fisheries Cluster, Institute for the Oceans and Fisheries, University of British Columbia, Vancouver, British Columbia, Canada; 2 UNEP World Conservation Monitoring Centre, Cambridge, United Kingdom; 3 NF-UBC Nereus Program, Institute for the Oceans and Fisheries, University of British Columbia, Vancouver, British Columbia, Canada; 4 Aboriginal Fisheries Research Unit, Institute for the Oceans and Fisheries, University of British Columbia, Vancouver, British Columbia, Canada; 5 Department of Zoology, University of Cambridge, Cambridge, United Kingdom; GEOMAR Helmholtz Center for Ocean Research, GERMANY

## Abstract

Studies have demonstrated ways in which climate-related shifts in the distributions and relative abundances of marine species are expected to alter the dynamics and catch potential of global fisheries. While these studies assess impacts on large-scale commercial fisheries, few efforts have been made to quantitatively project impacts on small-scale subsistence and commercial fisheries that are economically, socially and culturally important to many coastal communities. This study uses a dynamic bioclimate envelope model to project scenarios of climate-related changes in the relative abundance, distribution and richness of 98 exploited marine fishes and invertebrates of commercial and cultural importance to First Nations in coastal British Columbia, Canada. Declines in abundance are projected for most of the sampled species under both the lower (Representative Concentration Pathway [RCP] 2.6) and higher (RCP 8.5) emission scenarios (-15.0% to -20.8%, respectively), with poleward range shifts occurring at a median rate of 10.3 to 18.0 km decade^-1^ by 2050 relative to 2000. While a cumulative decline in catch potential is projected coastwide (-4.5 to -10.7%), estimates suggest a strong positive correlation between the change in relative catch potential and latitude, with First Nations’ territories along the northern and central coasts of British Columbia likely to experience less severe declines than those to the south. Furthermore, a strong negative correlation is projected between latitude and the number of species exhibiting declining abundance. These trends are shown to be robust to alternative species distribution models. This study concludes by discussing corresponding management challenges that are likely to be encountered under climate change, and by highlighting the value of joint-management frameworks and traditional fisheries management approaches that could aid in offsetting impacts and developing site-specific mitigation and adaptation strategies derived from local fishers’ knowledge.

## Introduction

Theoretical and empirical evidence of the influence of anthropogenic climate change on the productivity and ecology of marine ecosystems has been witnessed globally, spanning tropical, temperate, and polar regions [1–4]. Over 90 per cent of observed warming has accumulated within marine ecosystems, with sea surface temperature (SST) projected to warm between 0.6°C and 2.0°C by the end of the 21^st^ century [5]. Recent studies argue that climate change and the resulting range of biological responses, such as altered species distributions [2,3,6,7], phenology [4], physiology [8], and marine biodiversity [9,10], are likely to impact fisheries [1,11,12] and the societies that depend upon them [13,14]. In particular, projected changes in fisheries catch potential [12] could result in, or exacerbate, socio-economic impacts on fisheries through reduced food and economic security [15,16].

While available climate change impact assessments on marine fisheries frequently focus on large-scale commercial sectors, few efforts have been made to generate quantitative scenarios of potential effects on small-scale fisheries that exhibit social, cultural, and economic dependence on marine ecosystems. Small-scale commercial and subsistence fisheries are of critical importance to food security and poverty alleviation worldwide [17]. However, these fisheries are also susceptible to climate change impacts through shifts in distributions, compositions and potential yields of fish stocks.

First Nations situated along the Pacific coast of Canada are representative of Indigenous communities whose small-scale subsistence and commercial fishing practices and diversified harvest and storage of marine resources, such as salmon, have played essential roles in the development of their cultural complexity [18–21]. These Nations have demonstrated exemplary resilience in the face of anthropogenic and environmental change for millennia, having occupied this region for more than 10,000 years [22,23]. They have therefore attained considerable experience in accommodating environmental change and interpreting ecological indicators and relationships through traditional ecological knowledge (TEK)[24–27]. However, given the intrinsic importance of marine ecosystems to coastal First Nations, as cited extensively in the archaeological and anthropological literature [28–31], unprecedented climate change [32] poses a considerable threat to First Nations’ food and economic security, cultural practices, and spiritual values through fisheries [27,33]. Many First Nations have noted significant climate-related impacts manifesting in decreased availability of traditional foods through declining abundances, altered growth and migration patterns, and reduced predictability previously established through TEK and traditional phenological knowledge (TPK; [24,27,34,35]). Thus, it is necessary to improve our understanding of challenges that are likely to be faced increasingly by Indigenous peoples under climate change. This endeavor can be supported by identifying scenarios likely to occur under climate change, thereby allowing for the development and implementation of appropriate mitigation and/or adaptation strategies that are informed by First Nations’ TEK.

Increasingly, scenario-based assessments have enabled us to examine questions concerning the impacts of climate change on marine fisheries [15,36]. Plausible scenarios constructed from expert knowledge have been employed by the Intergovernmental Panel on Climate Change (IPCC) to serve as a framework for investigating key questions and for engaging in discussions regarding mitigation and adaptation prospects [37]. As models have been used in fisheries science as necessary tools to address uncertainty and produce defensible management strategies from existing knowledge [38], the integration of climate projections with fisheries models has enabled development of useful scenarios outlining potential effects on marine ecosystems and fisheries [39,40]. In particular, species distribution models (SDMs) have been used in conjunction with climate projections to explore trends in distributional shifts [7,41,42], species turnover [43], and corresponding impacts on the biophysics and economics of global fisheries [15,16] under climate change.

Studies have qualitatively explored factors influencing the resilience of First Nations in British Columbia [24,44], highlighted the potential application of TEK in understanding and responding to climate change [45,46], and produced frameworks for assessing the vulnerability of remote Indigenous communities to climate change [47,48]. This study progresses a step further by projecting plausible quantitative scenarios of how coastal First Nations’ key marine resources might be affected by climate change, identifying corresponding fisheries management challenges that would likely emerge under these scenarios, and discussing potential adaptive responses to these specific challenges informed by traditional fisheries management strategies. To achieve this, a SDM driven by outputs from global coupled ocean-atmosphere-biogeochemistry earth system models was used to project changes in potential yields of species targeted by coastal First Nations’ fisheries under climate change. With reference to these results, we discuss potential climate-resilient pathways that draw from examples of First Nations’ traditional fisheries management strategies.

## Methods

### Case study areas

While referred to collectively as First Peoples of the Pacific Northwest Coast [49,50], First Nations residing along coastal BC vary with respect to culture, traditions and diet associated with their fishing practices, making it inappropriate to assess climate-related impacts on First Nations as if they were a single, homogenous entity [34,51]. Therefore, First Nations were purposively selected from each of the seven coastal administrative regions defined by the BC First Nations Fisheries Council (FNFC), forming a sample of groups with diverse marine resources, geographical locations, territorial sizes, and treaty statuses. Ultimately, 16 of 78 First Nations identified near BC’s coastline were selected, 12 of which fall under three overarching councils negotiating in the BC treaty process: the Council of the Haida Nation, the Tsimshian Nations Treaty Society, and the Maa-nulth First Nations. For the purpose of this study, these 16 Nations are referred to collectively under their respective treaty councils, with estimated populations recorded as of December 2013 **(**Table 1) [52, 53]. The approximate sizes of the domestic fishing areas were derived from the Statement of Intent boundary shapefile using *ArcGIS* (see S1 Methods).

**Table 1:** Sample of First Nations included in this study and their respective regions and treaty groups.

FIRST NATIONS	ADMINISTRA-TIVE REGION	ECO-REGION	TREATY GROUP	REG.POP.	EST. SIZE OF DFA (sq. km.)
Gitga’at, Kitasoo/Xaixais, Kitselas, Kitsumkalum, and Metlakatla First Nations	North Coast	Hecate Strait/ Dixon Entrance	Tsimshian First Nations	3,508	8,520
Skidegate Band Council, Old Massett Village Council	Haida Gwaii	Haida Gwaii	Council of the Haida Nation	4,566	74,235
Heiltsuk First Nation	Central Coast	Central Coast	Independent	2,362	10,800
‘Namgis First Nation	North Vancouver Island and Mainland Inlets	Central Coast	Independent	1,787	2,615
Huu-ay-aht, Ka:’yu:’k’t”h’ / Chek’tles7et’h, Toquaht, Uchucklesaht, and Ucluelet First Nations	West Coast Vancouver Island	West Coast Vancouver Island	Maa-nulth First Nations	2,231	18,870
Tla’amin (Sliammon) First Nation	South Island and Mainland Inlets	Strait of Georgia	Independent	1,035	6,087
Tsawwassen First Nation	Lower Mainland	Strait of Georgia	Independent	342	1,215

The biogeographical characteristics of coastal BC are characterized by considerable terrestrial and marine-based diversity due to a temperate climate and coastal upwelling system that generates nutrient-rich waters [22]. These conditions yield highly productive environments with complex marine food webs and diverse coastal landscapes, from rocky intertidal zones, shallow rocky reefs, kelp forests, sandy nearshore areas, and estuarine ecosystems [22]. While these regions share similar species assemblages and are characterized by comparable environmental conditions (e.g., oceanic currents and wind-driven upwelling) [54], the FNFC’s administrative regions intersect with five distinct ecological regions: the North Coast, comprising the Hecate Strait and Dixon Entrance; Haida Gwaii, which includes the waters surrounding the islands; the Central Coast, including Queen Charlotte Sound, Queen Charlotte Strait, and the southern tip of Hecate Strait; the Strait of Georgia; and the west coast of Vancouver Island (WCVI) [55–59]. First Nations are likely exposed to different climate-related impacts on fisheries due to the differing ecological and biogeographical characteristics of these regions and to differing traditional and commercial harvests.

First Nations’ domestic fishing areas (DFAs) were derived from the approximate Statement of Intent (SOI) boundaries registered with the BC Treaty Commission as of October 2004, which were converted to 0.5° latitudinal by 0.5° longitudinal grid-cells to correspond with downscaled global climate model grids (S1 Methods). While these boundaries do not signify the full extent of territory previously used by First Nations, particularly with respect to the sharing of resources between communities [44,51,60,61], they serve to illustrate approximate areas requested by First Nations for FSC and commercial fishing purposes.

### Selection of species

A sample of culturally and commercially important species was identified from peer-reviewed literature, government and non-governmental organisations’ reports, treaty agreements, and First Nations’ reports. Ninety-eight species—comprising marine and diadromous fish, shellfish, and invertebrates—were selected (sample of species harvested for food, social, and ceremonial [FSC] purposes summarized in Table 2 [30,62–69]; full list of commercial and FSC species available in S1 Table). This list is not intended to be a complete representation of the resources used by coastal First Nations or to indicate relative importance, but a summary restricted to a sample of key marine fishes, shellfish and invertebrates harvested by First Nations, as documented in the available literature. While also contributing to First Nations’ traditional harvests, marine mammals, plants and birds were not the focus of this study.

**Table 2:** Sample of species harvested by First Nations for food, social and ceremonial (FSC) purposes, ordered alphabetically [30,62–69].

COMMON NAME(S)	SCIENTIFIC NAME(S)	EXAMPLES OF FISHING METHODS
Abalone, northern	*Haliotis kamtschatkana*	By hand or spear
Chitons	*Katharina tunicate*, *Cryptochiton stelleri*	By hand
Clams, intertidal (butter, manila, Pacific littleneck, varnish)	*Saxidomus gigantea*, *Venerupis philippinarum*, *Protothaca staminea*, *Nuttallia obscurata*	By hand
Clam, Pacific razor	*Siliqua patula*	Digging
Crab spp. (Dungeness, Pacific rock, tanner, purple shore, green)	*Metacarcinus magister*, *Cancer productus*, *Chionoecetes bairdi*, *Hemigrapsus* spp.	Handpicking, traps, gaffing, dip net, ring net
Dogfish, spiny	*Squalus suckleyi*	By hook and line
Eulachon (oolichan)	*Thaleichthys pacificus*	By net (driftnet, bag net); rake
Flounder and soles	*Pleuronectidae*	Hook and line; traps; seine net
Halibut, Pacific	*Hippoglossus stenolepis*	Hook and line
Herring, Pacific (including roe)	*Clupea pallasii pallasii*	Spawn on kelp; seine; gillnet; dip net; herring rake; hand picking
Lingcod	*Ophiodon elongatus*	Hook and line; jigging in shallow waters; trolling
Mussels (Pacific blue, northern horse)	*Mytilus trossulus*, *Modiolus modiolus*	By hand
Prawn	*Pandalus platyceros*	By trap
Rockfish	*Sebastes* spp., *Sebastolobus* spp.	Hook and line; Jigging in shallow waters; trolling
Sablefish (black cod)	*Anoplopoma fimbria*	Hook and line; traps
Salmon (sockeye, chum, pink, Chinook, coho)	*Oncorhynchus* spp.	Traps, weirs, beach seines; trap nets; fish wheels; seine; hook and line; dip or gillnets; spear
Scallops (weathervane, spiny pink, rock)	*Chlamys hastata*, *Patinopecten caurinus*, *Crassadoma giganteus*	Collect by hand
Sea cucumber	*Parasthichopus californicus*	Collect by dive
Shrimp	*Pandalus* spp., *Pandalopsis* spp.	Trap
Sturgeon, white	*Acipenser transmontanus*	Harpoon; weir; set or trawl net
Urchins	*Strongylocentrotus* spp.	Spear or collect by hand

### Projecting effects of climate change on First Nations’ fisheries

A dynamic bioclimate envelope model (DBEM) [39,70] was used to model changes to the distributions and relative abundances of the studied species under two climate change scenarios. Current (1971–2000) species’ distributions were obtained using an SDM developed by the *Sea Around Us* Project [49,71]. The model determines distributions of marine fishes and invertebrates by employing a set of filters including: (i) presence in FAO area(s); (ii) latitudinal range; (iii) range-limiting polygons; (iv) depth range; (v) habitat preferences; and (vi) the effect of ‘equatorial submergence’ (for detailed methodology, please refer to [71]). Data for these filters were primarily derived from FishBase [49,72], SeaLifeBase [73] and the Encyclopedia of Life [74,75], which were supplemented or cross-checked with data collected from the Department of Fisheries and Oceans Canada (DFO) reports, and peer-reviewed literature. For species with limited life history records, data for species within the same genus were used. Range-limiting polygons were produced using known latitudinal and longitudinal ranges and the resulting distributional maps were cross-checked with AquaMaps [76], the IUCN Red List of Threatened Species [77], and FAO’s Aquatic Species Distribution Viewer [78] (see S1 Data).

Based on modelled current distributions, the DBEM simulates changes in the relative abundance of each species in each 0.5° latitudinal x 0.5° longitudinal cell of the ocean. By overlaying the current predicted relative abundance on modelled ocean properties from Earth System Models for the contemporary period (1971–2000), we identified species’ preferences to environmental conditions that are defined by sea water temperature (surface and bottom), salinity, sea ice concentration, bathymetry and habitat types (e.g., estuaries, seamounts, shelf). Species’ carrying capacities (*K*) in each cell (*i*) are positively related to inferred environmental preferences and the environmental conditions. Changes in relative abundance (*Abd*_*i*_) in each cell (*i*) at each time step (*t*) were simulated based on the following algorithm (see Cheung et al. 2011 for details):

$$\frac{d(Abd_{i})}{dt}=\;\sum_{j=1}^{N}G_{i}+L_{ji}+I_{ji}$$ (1)

where *Abd*_*i*_ is the relative abundance of cell *i*, *G* is the intrinsic population growth, and *L*_*ji*_ and *I*_*ji*_ are settled larvae and net migrated adults from surrounding cells (*j*), respectively [9,79]. Recruitment within the model thereby operates as a function of larval dispersal, settlement, and mortality, which is temperature dependent. However, the DBEM does not aim to represent the natural short-term (inter-annual) variability of recruitment; instead it aims to represent the long-term mean changes in production of the population. The intrinsic growth rate of a population is calculated as

$$G_{i}=r\cdot Abd_{i}\cdot (1-\frac{Abd_{i}}{K_{i}})$$ (2)

where *r* is the intrinsic rate of population increase and *K*_*i*_ is the population carrying capacity for cell *i* that is expressed as a function of the habitat suitability, and theoretical unfished biomass of the cell. An advection-diffusion-reaction model is used to account for larval dispersal according to ocean conditions, while adult abundances diffuse following a gradient of habitat suitability [9]. Further details regarding these methods were previously published by Cheung et al. (2009, 2010; [9,12,42]).

Model simulation was driven by projected changes in ocean properties under two climate change scenarios: RCP 8.5 and 2.6, representing the high and low greenhouse gas emission scenarios respectively [37,80,81]. Ocean properties—including sea surface temperature, sea bottom temperature, salinity, oxygen concentration, surface advection, and net primary production—were projected from NOAA’s Geophysical Fluid Dynamics Laboratory’s IPCC-class earth system model (GFDL ESM2M; [82]). Based on the outputs from the DBEM, we calculated changes in species’ relative abundances, distributions, and richness, as well as corresponding changes in relative catch potential using species’ relative abundances and projected changes in primary productivity for each cell (methods based on [70] and [42]).

The latitudinal centroid (*LC*) of each species was calculated as

$$LC=\frac{\sum_{i=1}^{n}Lat_{i}*Abd_{i}}{\sum_{i=1}^{n}Abd_{i}}$$ (3)

where *Lat*_*i*_ is the latitudinal centre of the spatial cell (*i*) and *n* is the total number of cells where abundance is greater than 0 [9,83]. The range shift, in kilometers, was then calculated from the difference between the latitudinal centroid of the projected and reference years as follows [70]:

$$\mathrm{Distance shifted}\;\left(\mathrm{km}\right)=\;\left(LC_{m}-LC_{n}\right)\frac{\pi }{180}\;\times \;r$$ (4)

where *r* = 6378.2 km, the approximate radius of the earth, and *LC*_*m*_ and *LC*_*n*_ are the latitudinal centroids in years *m* and *n*. Median values were used to estimate range shifts by taxonomic group in order to remove anomalies. The relative change in catch potential (%) was estimated from mean inferred current abundance (1971–2000), change in net primary production, and range area, as represented by the following algorithm,

$$\Delta \;{\mathrm{Relative Catch Potential}}_{t}\;\left(\%\right)=\frac{\sum \;{\mathrm{Abundance}}_{t}\;\times \;P_{i,t}\;\times \;A_{i,t}}{\sum \;{\mathrm{Abundance}}_{t0}\;\times \;P_{i,t0}\;\times \;A_{i,t0}}\;\times \;100\%$$ (5)

where *P* and *A* are the net primary productivity and area of cell *i* at time step *t*, respectively [12,36]. Due to limited landings data, the results were reported as percent change in relative catch potential. Changes in species richness were inferred by modifying relative average abundance data for the projected (2041–2060) and reference (1991–2010) periods to reflect presence (1) or absence (0) within a given cell and summed to yield species richness for each cell within the area of interest. The difference was then obtained between the projected and reference periods. The threshold used to assess species’ presence or absence was 0 (Values > 0 were assigned a value of 1), which represents an extremely conservative estimate given that species’ thresholds would differ depending on the spatial density and dynamics of the populations [84].

Relative changes in commercial catch potential (%) were calculated for each 0.5° longitudinal x 0.5° latitudinal grid-cell situated in BC’s waters within Canada’s exclusive economic zone (EEZ) using 20-year average abundances for 2050 (2041–2060) relative to 2000 (1991–2010) obtained from the DBEM simulations. Species were aggregated by commercial fishery, noting the proportion of species included in the analysis relative to those included in each fishery’s quota (S2 Table). Regional impacts on FSC fisheries were estimated by using First Nations’ DFAs as outlined by the SOI boundaries submitted to the BC Treaty Commission, with the change in relative catch potential calculated for each species within each DFA.

Annual landed values by taxon or fishery (e.g., herring spawn-on-kelp) between 2001 and 2010 were obtained from the BC Ministry of Agriculture’s annual reports and averaged to provide mean annual landed revenue [85–89]. Projected changes in relative catch potential for each commercial fishery under RCPs 2.6 and 8.5 were subsequently used to derive an estimate of changes in mean annual landed revenue (in 2010 CAD) given the approximate proportion of licenses held by First Nations [90](S3 Table). These changes in landed revenue were calculated using the following equation:

$$\Delta R=R\;\times \;\left(\frac{\Delta \mathrm{CP}}{100}\right)$$ (6)

where **Δ*R*** is the projected change in First Nations’ mean annual landed revenue by 2050, ***R*** represents First Nations’ estimated current mean annual landed revenue (2001–2010), and **Δ*CP*** represents the projected per cent change in relative catch potential by 2050. Data outlining landed revenue in the Heiltsuk commercial intertidal bivalve fishery and First Nations’ participation in the commercial green sea urchin fishery, tuna fishery, and groundfish trawl and hook and lines fisheries were not available, and were therefore not included in the estimates of changes in landed revenue in the EEZ.

### Sensitivity analysis

Results obtained by Jones and Cheung [42] for two additional SDMs, Maxent [91] and AquaMaps [92], were used to analyse the sensitivity of relative catch potential and latitudinal range shifts obtained under RCP 8.5 to alternative SDM approaches and their underlying assumptions. In contrast with the DBEM’s discriminative approach that combines mechanistic and statistical tools, Maxent and AquaMaps use statistical methods to produce bioclimate envelopes that represent species’ relative habitat suitability. Species’ current distributions were determined by associating presence-only data with 30-year averaged environmental data (1971–2000). Results from AquaMaps and Maxent were available for 31 and 33 of the 98 species included in this study, respectively.

## Results

### Impacts on species’ relative abundance, distribution and richness

Declines in relative abundance were projected by 2050 (relative to 2000) for the majority of sampled species (87 of 98 spp.) within British Columbia’s marine environment under both scenarios of climate change, with evidence of latitudinal and regional trends. In contrast, white sturgeon (*Acipenser transmontanus*) and Pacific sardines (*Sardinops sagax*) were projected to increase in abundance within BC’s marine environment under both scenarios, while manila clams (*Venerupis philippinarum*) were projected to increase in abundance by 14.5% under RCP 8.5, The remaining species (8 spp.) showed little change, with obtained projections oscillating around the baseline (-8.3 to +4.6%)(S4 Table).

**Fig 1:**
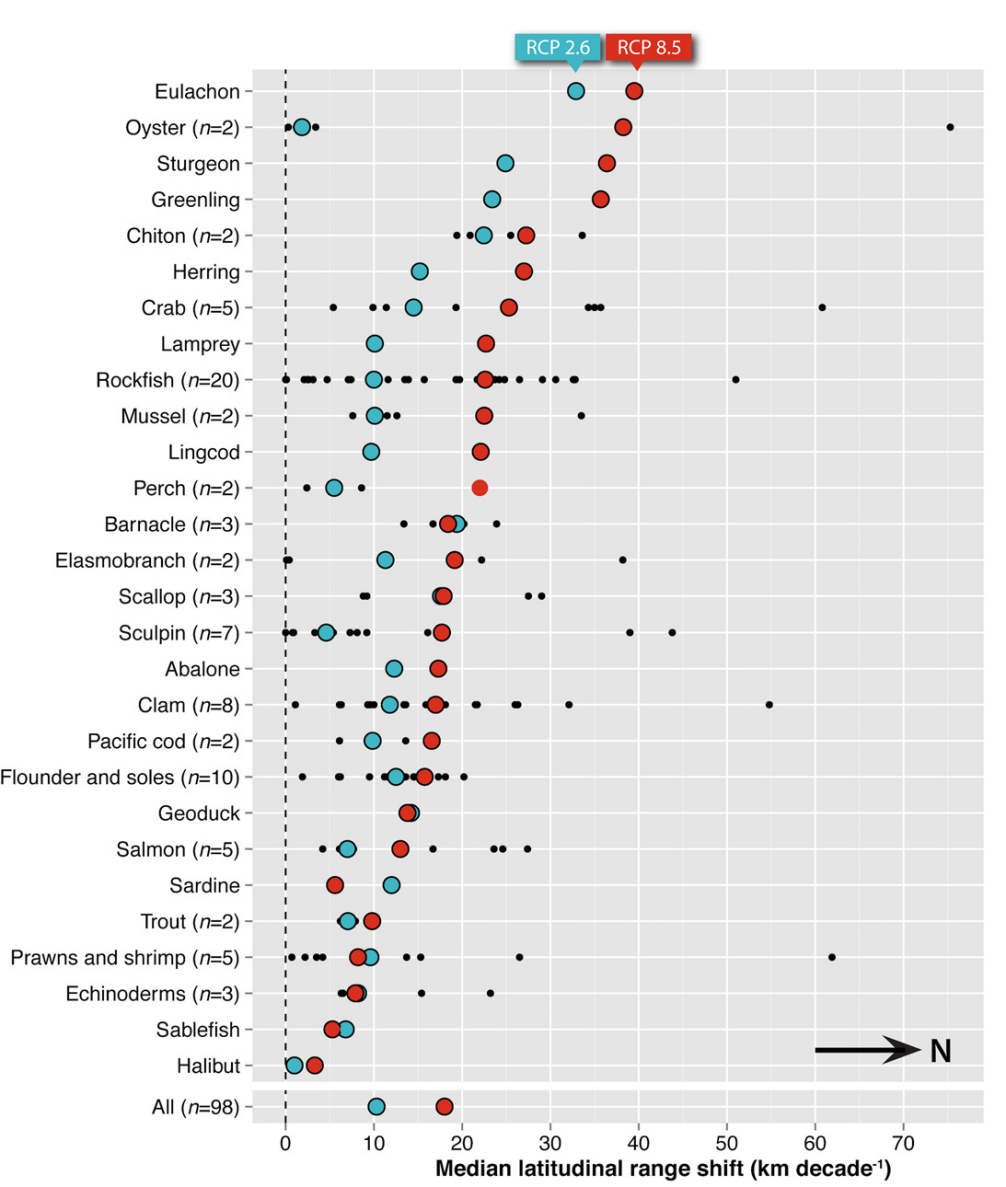
Projected median latitudinal range shifts (km decade^-1^) by taxonomic group or species. Projections used an average 20-year latitudinal centroid centred on 2050 relative to that centred on 2000 under the lower (blue; RCP 2.6) and upper (red; RCP 8.5) scenarios of climate change. Where applicable, black dots represent the results for each species that were used to determine the aggregated median values.

A poleward range shift in mean abundance by 2050 was projected for all species examined, with an estimated total median rate of 10.3 to 18.0 km decade^-1^ under the lower and upper scenarios of climate change respectively (Fig 1). A median rate of 10.1 to 17.8 km decade^-1^ was projected for demersal species, while pelagic species were projected to move polewards at a median rate of 13.6 to 33.3 km decade^-1^. Rates tended to increase under the upper scenario of climate change relative to the lower scenario (68 of 98 spp.). Most of the remaining species (30 spp.) exhibited similar rates of poleward movement under both scenarios (i.e., within 7 km decade^-1^ for 26 of 98 spp.), while only a few (4 spp.) exhibited slower rates of poleward movement (S5 Table).

While most species were projected to decline in relative abundance and to shift polewards relative to BC’s marine environment by 2050, species richness along coastal BC was projected to change little under either scenario of climate change. The absolute number of species that occurred within each cell (i.e., with a habitat suitability of greater than zero) reflected gains or losses of up to 3 species per cell. Similar latitudinal patterns to those observed for relative abundance and distribution were again evident, with greater losses in species richness likely to occur towards the southern coast of British Columbia, falling primarily between 48°N and 51°N. Notably, changes in species richness were projected off the coasts of Alaska and California, which mark the northern and southern latitudinal extents of many of the species’ distributions included in this analysis.

### Impacts of climate change on First Nations’ fisheries

#### Impacts on commercial catch potential and revenue

**Fig 2:**
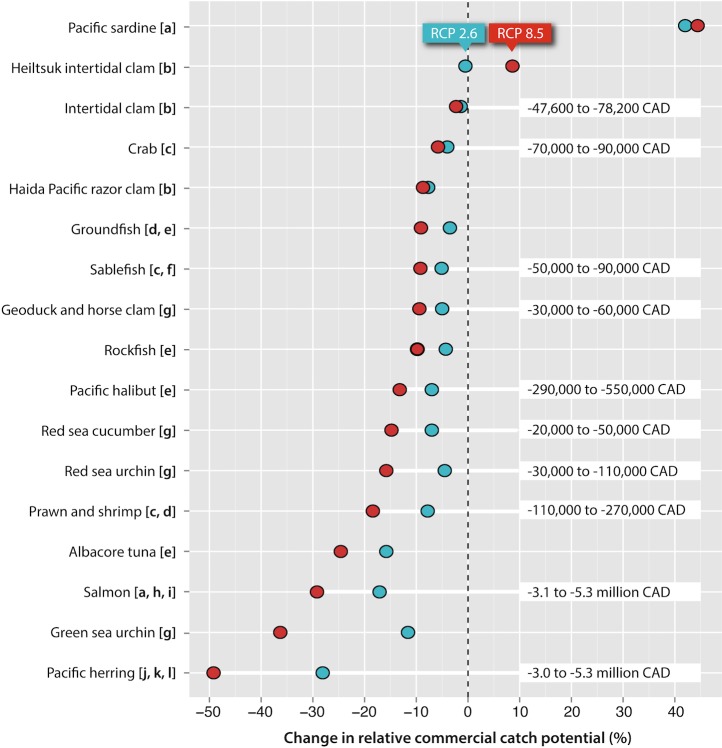
Projected change in relative catch potential by commercial fishery with known First Nation participation. Changes were calculated using 20-year average catch potential for 2050 relative to 2000 within British Columbia’s marine environment. Projections reflected the lower (blue; RCP 2.6) and upper (red; RCP 8.5) ranges of climate change. Values on the right reflect conservative cumulative estimates of impacts on First Nations’ commercial fisheries revenue (median values for 2001–2010 in CAD). Letters represent the type of commercial fishery: [a] seine, [b] hand, [c] trap, [d] trawl, [e] hook and line, [f] longline, [g] dive, [h] troll, [i] gill net, [j] roe herring, [k] spawn on kelp, and [l] bait (see S2 Table).

With two exceptions, modest to severe declines in catch potential were suggested for all commercial fisheries with known First Nation participation (15 out of 17 spp.) under both scenarios of carbon emissions (Fig 2; S2 Table). Estimates placed the Pacific herring commercial fisheries, comprising roe herring, spawn-on-kelp, and food and bait fisheries, as likely to experience the greatest relative impact under both climate change scenarios, with declines in catch potential ranging between 28.1 and 49.2% across coastal BC. Collectively, salmon (*Oncorhynchus* spp.) were projected to exhibit cumulative declines in catch potential of 17.1% to 29.2% within BC’s marine environment.

In contrast, an increase in the relative abundance of warmer-water species was projected to lead to new or increased opportunities for commercial harvests by 2050. For instance, catch potential for the commercial Pacific sardine (*Sardinops sagax*) fishery is likely to expand in abundance, with estimates ranging from 42.0 to 44.4% relative to the current baseline within BC’s marine environment. At a more localized scale, estimates indicated that the Heiltsuk intertidal clam fishery for manila clams (*Venerupis philippinarum*) and Pacific littleneck clams (*Protothaca staminea*) may experience slight gains in catch potential under the upper climate change scenario (+8.6%), or experience relative neutrality under the lower scenario (-0.5%).

Conservative estimates of lower- and upper-thresholds of regional losses in annual landed revenue to a selection of First Nations’ commercial fisheries ranged between 6,720,000 and 11,820,000 CAD, translating to a 16.4% to 28.9% reduction in total landed revenue by 2050 based on 2001–2010 median values (Table 3). Collectively, the commercial herring and salmon fisheries contributed approximately 74% of First Nations’ landed revenue in 2003 [90], and are expected to comprise the majority of the decline in revenue under climate change (i.e., between 89.3% and 90.2%). These estimates do not account for impacts on auxiliary industries, such as fish processing and marketing, nor do they account for potential opportunities attained through an increased abundance of lower-latitude species. In particular, commercial sardine fisheries, for which First Nations held 50 per cent (*n =* 25) of allocated licenses between 2012 and 2015 [93], were projected to see increases within BC’s waters under both climate change scenarios.

**Table 3:** Estimated upper and lower thresholds of impacts on mean annual landed revenue (2001–2010 CAD) for a sample of First Nations’ (FNs’) commercial fisheries. Values represent estimated changes under the lower (RCP 2.6) and upper (RCP 8.5) climate change scenarios [85–90].

						Climate change scenarios			
	Estimated annual commercial revenue (2003)[adapted from 90])			Mean annual commercial revenue (2001–2010)		RCP2.6		RCP8.5	
	Landed revenue ($ millions, avg. 1999–2002)	Estimated FNs’ revenue ($ millions CAD)	Proportion (%)(FN-held and operated)	Mean annual landed revenue ($ millions, 2001–2010 CAD)	Estimated FNs’ annual landed revenue ($ millions CAD)	Change in catch potential (%)	Loss in FNs’ annual landed revenue (millions, 2001–2010 CAD)	Change in catch potential (%)	Loss in FNs’ annual landed revenue (millions, 2001–2010 CAD)
Salmon	49.9	20.5	41.1	43.8	18.0	-17.0	-3.06	-29.2	-5.26
Roe herring	37.6	10.2	27.1	22.9	6.2	-28.1	-1.74	-49.2	-3.06
Spawn-on-kelp	9.5	7.7	81.1	5.6	4.5	-28.1	-1.26	-49.2	-2.23
Halibut	41.6	3.9	9.4	44.5	4.2	-7.0	-0.29	-13.2	-0.55
Sablefish	27.9	1.2	4.3	23.8	1.0	-5.1	-0.05	-9.2	-0.09
Crab	26.7	1.3	4.9	34.7	1.7	-4.0	-0.07	-5.8	-0.09
Prawn and shrimp	30.3	1.2	4.0	34.0	1.3	-8.4	-0.11	-20.1	-0.27
Geoduck and horse clam	39.4	0.7	1.8	33.8	0.6	-5.0	-0.03	-9.4	-0.06
Red urchin	8.3	1.1	13.3	5.2	0.7	-4.5	-0.03	-15.8	-0.11
Sea cucumber	1.7	0.2	11.8	1.7	0.2	-7.0	-0.02	-14.8	-0.05
Intertidal clam	5.6	3.4	60.7	5.6	3.4	-1.4	-0.05	-2.3	-0.08
**TOTAL**	**363.7**	**51.6**	**18.4**	**254.7**	**40.9**	**TOTAL**	**-6.72**	**TOTAL**	**-11.82**

#### Impacts on food, social and ceremonial catch potential

**Fig 3:**
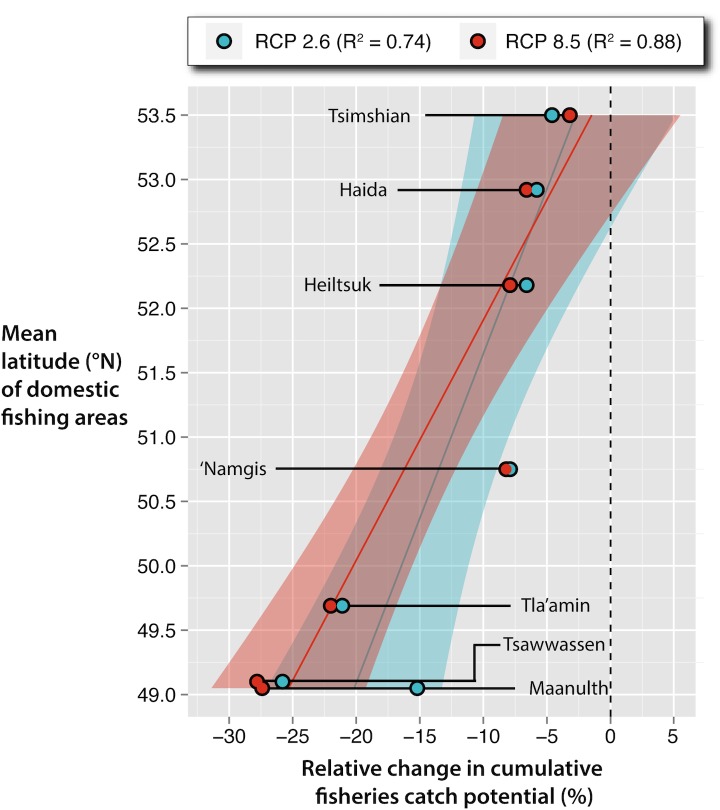
Relationship between latitude and cumulative change in catch potential (%) by 2050 from the baseline (0%) under the lower (RCP 2.6; blue) and upper (RCP 8.5; red) scenarios of climate change. Shaded bars represent 95% confidence intervals (data available in Table A in S1 Text).

**Fig 4:**
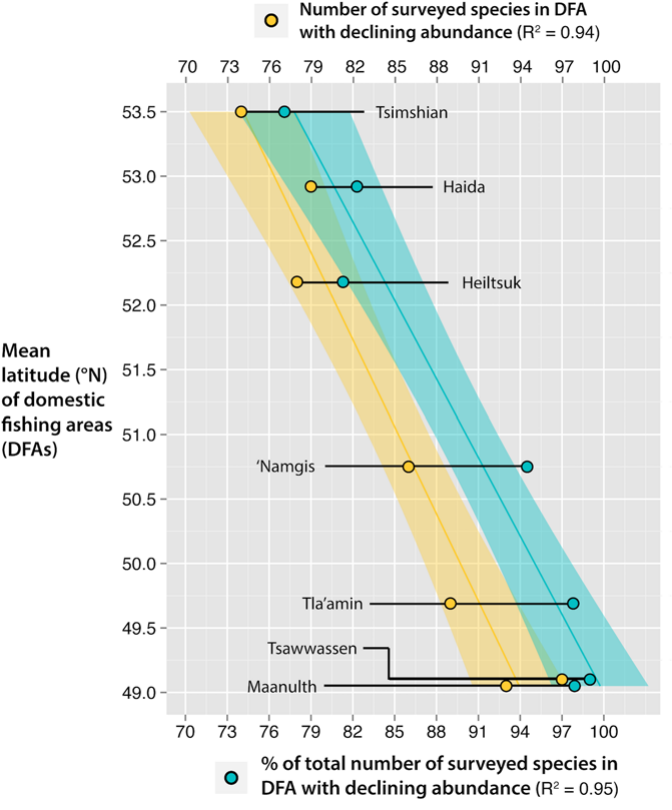
Correlation between latitude and (a) the number of species exhibiting declines in catch potential by 2050 (yellow) and (b) the percentage of the total number of surveyed species in the respective domestic fishing area exhibiting declining abundance by 2050 (blue). Declines are exhibited under both the lower (RCP 2.6) and upper (RCP 8.5) scenarios of climate change. Shaded bars represent 95% confidence intervals (data available in Table B in S1 Text).

Catch potential was projected to decrease for the majority of species harvested for FSC purposes under both scenarios of climate change across BC’s marine environment (S6 Table), with the change in relative cumulative catch potential by 2050 showing strong positive correlation with latitude (RCP 2.6: *R*^*2*^ *=* 0.744; RCP 8.5: *R*^*2*^ *=* 0.876; Fig 3). Furthermore, the number of species projected to decline by 2050 under climate change showed a strong negative correlation with latitude (RCP 2.6: *R*^*2*^ *=* 0.94; RCP 8.5: *R*^*2*^ *=* 0.95; Fig 4). These correlations strengthened for both relationships under the upper range of climate change (i.e., RCP 8.5).

While relative catch potential increased with latitude, all First Nations’ DFAs included in this analysis were projected to experience cumulative decreases in species abundance and corresponding catch potential, with less severe declines at higher latitudes. DBEM estimates suggested that the southern territories (Tsawwassen, Tla’amin, and Maa-nulth First Nations) will likely be exposed to a reduction in catch potential (between -15.2% and -27.8%) coinciding with both the upper and lower ranges of climate change. In contrast, the northern DFAs (Haida and Tsimshian First Nations) and those situated along the central or north-eastern coasts of Vancouver Island (Heiltsuk and ‘Namgis First Nations, respectively) were projected to experience lower relative reductions in relative catch potential for each territory, with estimates falling between -3.2 and -8.2%.

**Fig 5:**
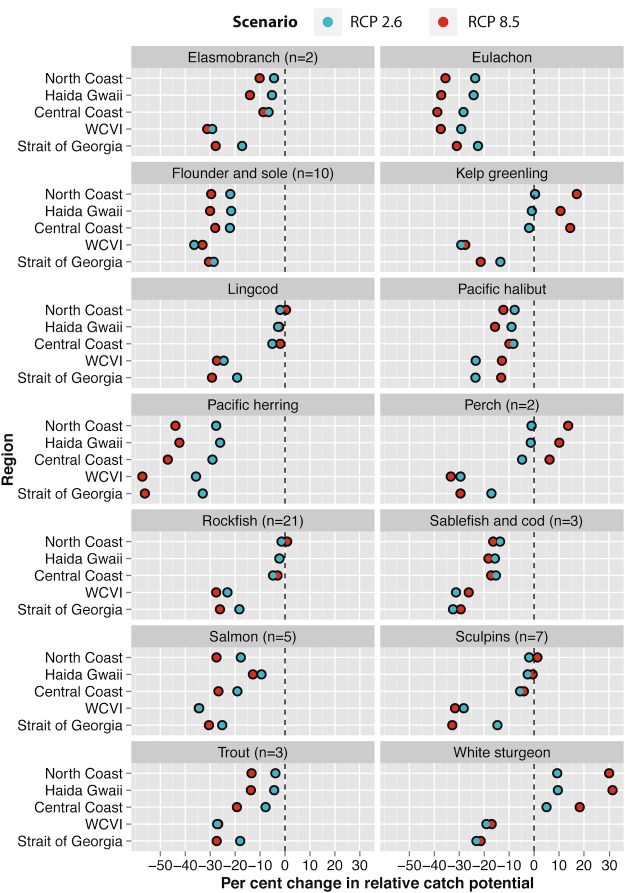
Change in relative catch potential by taxonomic group for each region, ordered from north to south. Regions in BC’s marine environment include the North Coast, Haida Gwaii, the Central Coast, the west coast of Vancouver Island (WCVI), and the Strait of Georgia. Projected changes in catch potential under the lower (RCP 2.6; blue) and upper (RCP 8.5; red) scenarios of climate change are denoted.

A few trends emerged when comparing changes across latitudes (Fig 5). For instance, projections for DFAs situated along the North and Central Coasts of British Columbia (Gitga’at and Haida, and Heiltsuk and ‘Namgis, respectively) indicated neutral or positive shifts in catch potential for white sturgeon (*Acipenser transmontanus*), kelp greenling (*Hexagrammos decagrammus*), and two species of perch (*Rhacochilus vacca* and *Brachyistius frenatus*) under both scenarios. While varying regionally, both scenarios also suggested either a slight cumulative decline or negligible change in catch potential for clams, rockfish, lingcod, and sculpins across the North and Central Coast DFAs.

In contrast, territories situated along the west coast of Vancouver Island (WCVI) and the Strait of Georgia tended towards more severe declines across taxonomic groups and species in comparison with those projected for the North and Central Coast regions. The exceptions to this included scallops and eulachon (*Thaleichthys pacificus*), which displayed more variable trends: for instance, catch potential for eulachon found in the Hecate Strait along the North Coast was projected to exhibit declines similar to those in the WCVI region.

Catch potential for Pacific herring (*Clupea pallasii*) was projected to decrease in all study areas under both climate change scenarios, with DBEM estimates ranging from -26.0 to -49.2%. The results from AquaMaps and Maxent corroborated this projected decline, but offered more conservative estimates (-2.2 to -17.0%). Similarly, declines in salmon, which are cultural keystone species for First Nations on the Pacific Northwest Coast [49], are anticipated for all regions under both scenarios.

#### Sensitivity analysis

**Fig 6:**
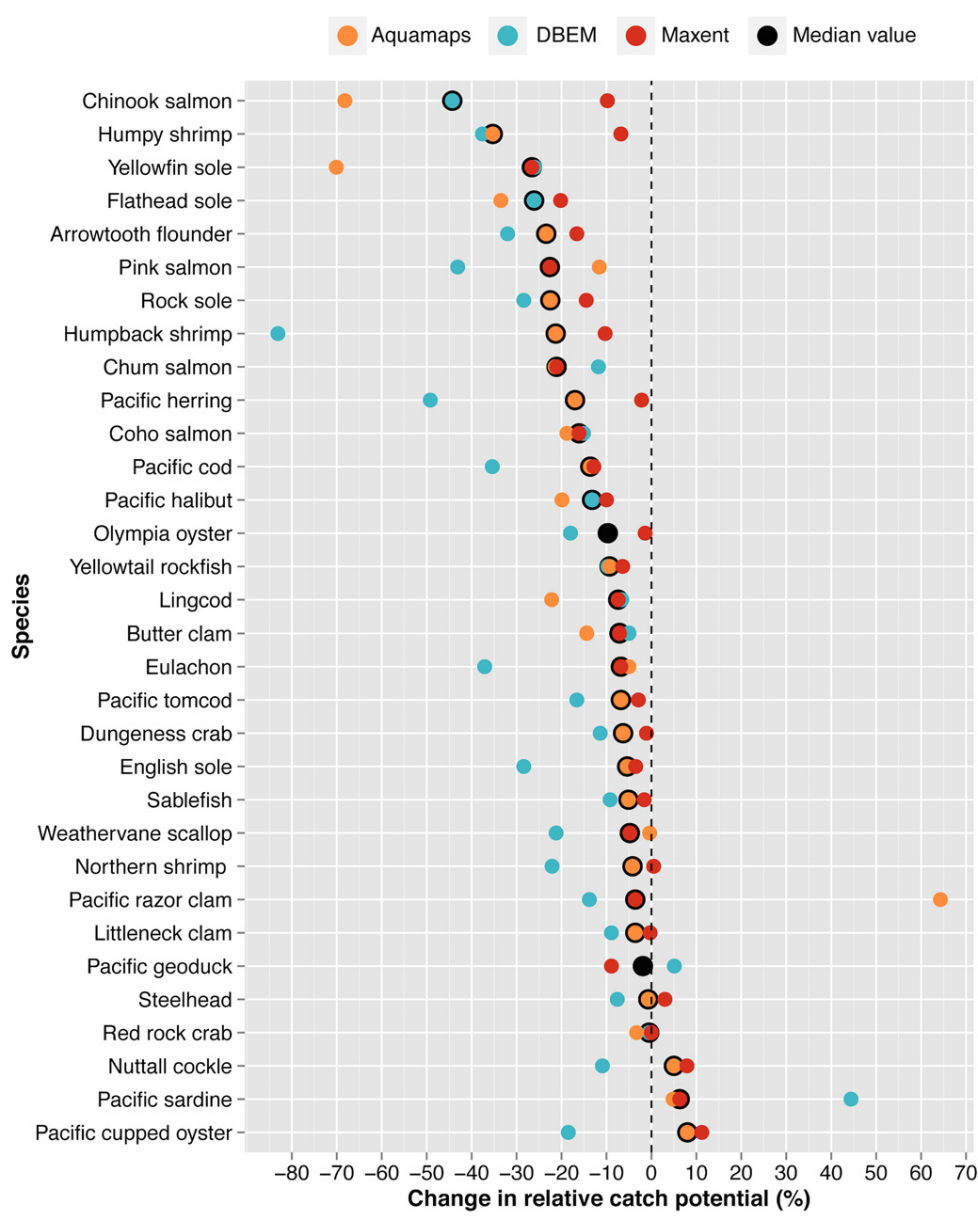
Multi-model ensemble examining the variability of projected change in relative catch potential by species (Table A in S2 Text).

**Fig 7:**
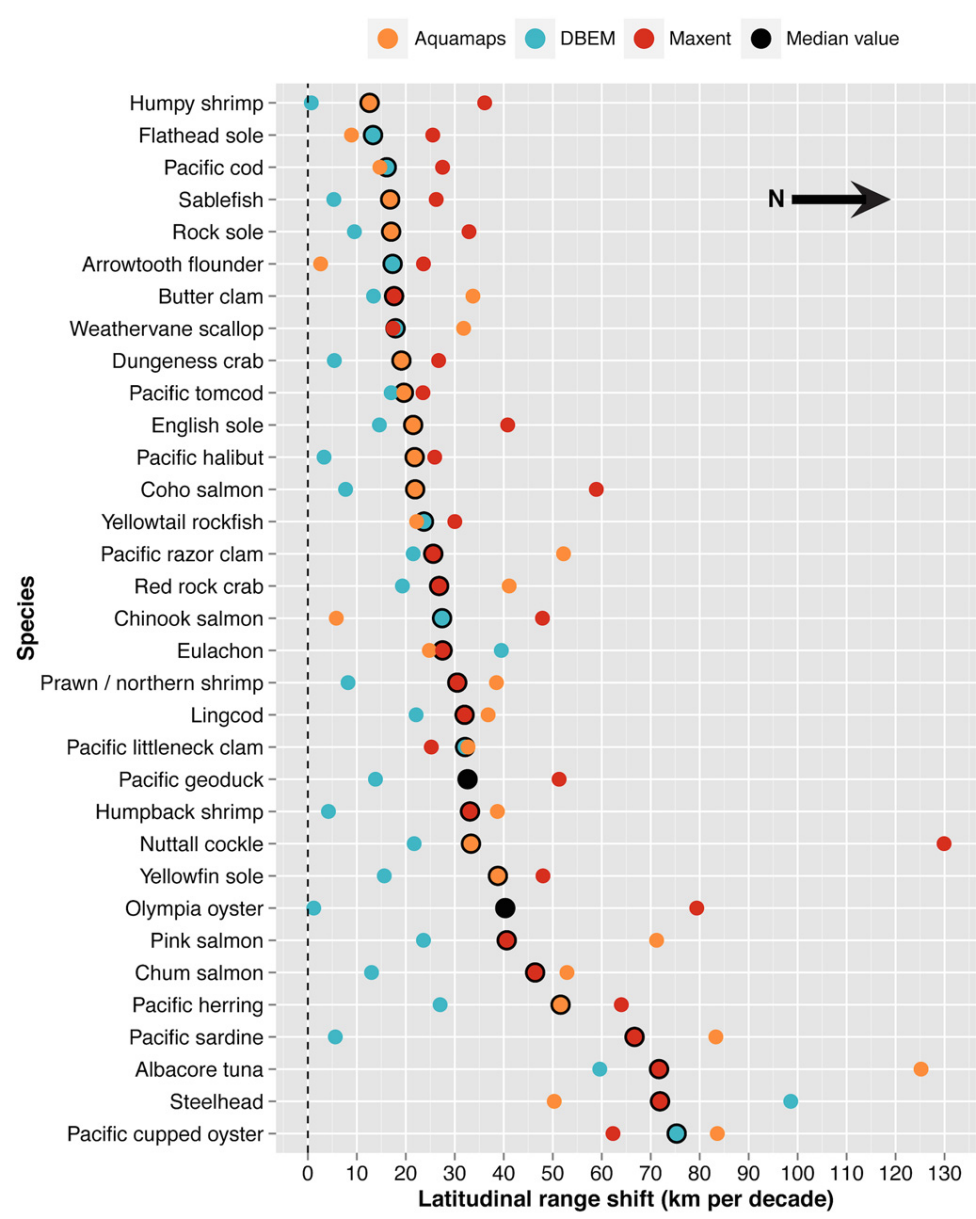
Multi-model ensemble examining the variability of projected latitudinal range shifts by species (Table B in S2 Text).

For most of the species tested, results from the multi-model ensembles suggested agreement in the direction of projected changes in relative catch potential (27 of 33 spp.; Fig 6; Table A in S2 Text) and latitudinal range shifts (33 of 33 spp.; Fig 7; Table B in S2 Text). Poleward range shifts were projected for all of the 33 species examined, agreeing with empirical and modeled results obtained in previous studies [12,36,94].

Projections for pelagic species, such as eulachon, Pacific sardines, Pacific herring, and albacore tuna, tended to exhibit the greatest inter-model variability for both latitudinal range shift and relative catch potential estimates (Tables A and B in S2 Text). In these instances, the DBEM suggested larger estimates than those produced by either of the other two models. For example, the DBEM estimated a 44.4% increase in the relative abundance of Pacific sardines versus the 4.8% and 6.3% increases projected by the other models, while the DBEM projected a decline of 37.1% for eulachon in contrast with the declines of 5.0% and 6.8% estimated by AquaMaps and Maxent, respectively. Given that many of these pelagic species are warmer-water species, theoretical and empirical evidence suggest a northward shift in abundance that would increase catch potential within BC’s waters [4,12]. However, due to highly migratory patterns, accurately determining distributional shifts for pelagic species at higher trophic levels can prove challenging [7].

## Discussion

### Implications for small-scale fisheries management

#### Spatial trends and regional offsets

An important concern highlighted by this study is the unequal distribution of relative losses in catch potential associated with regional ecological differences and poleward range shifts. In addition to coastwide declines in catch potential that are suggested when viewing cumulative effects on taxonomic groups (e.g., salmon and clams), species-based estimates reveal a stronger differential that correlates with latitude (i.e., impacts are less severe moving polewards).

While there is increasing support for establishing equitable resource sharing agreements between Nations [95] that might offer opportunities to offset such declines, the means by which such agreements could be achieved are uncertain [96]. Although appearing feasible in theory, three key issues arise from the prospect of applying these agreements to offset regional impacts: the scale of resource-sharing agreements, the extended time frame, and the requirement of reciprocity.

Firstly, resource sharing agreements have both existed and been discussed primarily in a localized context, such as a single river system [96], rather than at the regional level. For example, reciprocal agreements between First Nations historically allowed for shared use of a harvesting or processing site in exchange for another [97]. Although such resources sharing agreements could aid in increasing First Nations’ participation in commercial harvests for manila clams and geoducks along the North and Central Coasts, where their abundances are projected to sufficiently increase under climate change so as to offset other regional losses, the cost of travelling to these regions may offset the prospective benefits. Similarly, FSC harvests hinge upon the feasibility of accessing resources locally, making trans-regional trips unlikely to prove viable or desirable.

The remaining two issues of time frame and reciprocity are intertwined, and relate to the circumstances that facilitated resource exchanges. Many of the key traditional institutions that generated considerable adaptive capacity for First Nations primarily supported responses to seasonal or inter-annual fluctuations in resource abundance and availability through reciprocity [44], or operated as measures to prevent such long-term declines through spatial management [98], selective fishing [99], and forms of mariculture [100], among other strategies. Since reciprocal agreements were intended to provide insurance against future resource scarcity, they inherently pivoted on the exchange of resources or access rights. Given that the scenarios presented in this analysis suggest that all species are likely to exhibit a relative decline in abundance in the southern regions of British Columbia, few arrangements remain where species could be traded or access rights exchanged in accordance with this principle. An exception may occur for commercial fisheries, given the potential increased abundance of warmer-water species (e.g., sardines) that is projected to occur within BC’s waters under climate change.

#### Externalized impacts

In almost all circumstances, scenarios represented in this analysis illustrate outcomes where impacts are externalized, rather than internally resolved. For instance, redistributing access rights to fishing would serve to reallocate, rather than reduce, impacts. Given existing competition and conflict over key resources such as salmon and herring, it is unlikely that further reductions in catch potential associated with climate change will yield equitable outcomes.

Traditional clam beds serve as an ideal example of a method that could be applied to offset climatic impacts through internalized mechanisms, using local cultivation to generate increased productivity by enhancing native habitat rather than redirecting extraction efforts towards other regions or species. Clam gardens constructed in a manner akin to those situated near ancient settlements of the Northern Coast Salish and Laich-kwil-tach First Nations have been found to generate higher clam densities, biomass, and growth rates than non-walled beaches [100]. These benefits were observed for Pacific littleneck clams (*Protothaca staminea*) and butter clams (*Saxidomus giganteus*), two clams that are of cultural, economic, and ecological importance to the region [100]. Reinstating clam beds in First Nations’ territorial lands has been suggested as a means of simultaneously achieving local conservation and cultural objectives [101], and may thereby provide a politically and ecologically viable option for mitigating climate-related impacts.

#### Joint management frameworks

Management of salmon and herring stocks has been highly contentious due to the myriad of stakeholders who depend upon them, which include First Nations, recreational fisheries, and commercial fisheries. Therein, each group represents an additional layer of complexity: for example, the Fraser River system is home to nearly 100 First Nation communities, each of which depends upon their legal right to salmon harvests [96]. Aside from fulfilling societal needs, salmon serve as key ecological components of the Pacific Northwest Coast, functioning as the mechanisms by which nutrients are transferred from the ocean to freshwater and terrestrial ecosystems [102,103].

While the projections above suggest the inability to fully offset declines in salmon and herring through a redistribution of fishing effort achieved under resource access agreements exchanged by First Nations, attaining a state of collaboration between First Nations, DFO, and other sectors has the potential to yield beneficial ecological and political results, if implemented correctly [96]. Parallels exist between First Nations’ traditional fisheries management approaches and “modern” approaches (e.g., spatial management, mariculture, selective fishing, fishing closures), with differences arising primarily due to diverging worldviews (see S7 Table). By aligning analogous approaches, local application of traditional management strategies could provide opportunities to collaboratively engage in adaptive ecosystem-based management [104] and to coordinate efforts to attain conservation objectives [101].

Established examples of joint-management initiatives may serve to provide frameworks for furthering conservation objectives and implementing traditional management practices. For instance, Garner and Parfitt (2006) identified three key case studies of joint-management of salmon fisheries on the Pacific Northwest Coast that endeavour to integrate First Nations and TEK into fisheries management decisions [96]. For instance, the Nisga’a Nation have ensured their equal partnership in management by employing traditional fish wheel technology to monitor and assess stocks and by leveraging traditional ecosystem-based management practices that could be applied to plan long-term objectives and management approaches [105]. Through such joint-management regimes, traditional fisheries management strategies could be applied to advance localized research directives and to reduce impacts on stocks under unprecedented environmental change. Moreover, the risk of conflict over declining resources underlines the need to establish common and equitable ground to ensure successful joint management of fisheries, and to leverage collective expertise.

### Uncertainties and assumptions

#### Dynamic bioclimate envelope model (DBEM) and input parameters

The results obtained through this analysis are representative of species’ responses to changes in environmental suitability under climate change that have been predicted or observed in the scientific literature [4,7,42], and are likely conservative given the potential for cumulative impacts that are not included in the present analysis (e.g., acidification, overfishing, habitat loss, etc.)[70]. The DBEM was selected for the purpose of this study due to the model’s capacity to predict large-scale scenarios of ecological change for numerous species given limited biological and ecological data. This global model integrates population and dispersal dynamics, which aid in determining the biogeography of marine systems under scenarios of climate change [106]. Although this version of the DBEM does not explicitly incorporate additional factors such as the influence of trophic interactions, evolutionary change, or fishing pressure, the use of empirically obtained habitat preferences based on species’ current distributions indirectly accounts for a ‘realized’ niche that is constrained by trophic interactions [107]. While the resulting shift in species’ distributions from inclusion of trophic interactions differed only modestly [107], these interactions may play a larger role in coastal regions and at localized scales, potentially leading to greater variation in projected responses to changing environmental conditions.

SDMs are inherently contingent on our understanding of species’ environmental preferences and capacity to respond to environmental change. For this reason, model certainty is much higher for species of commercial importance, where extensive research has been conducted on life history traits, environmental tolerance limits, and the extent of distributions. A species’ capacity to adapt is sensitive to the time scale over which a change occurs, as well as the magnitude of the change in environmental conditions, both of which can be further exacerbated when faced with multiple stressors. Inclusion of cumulative pressures (e.g., changes in fishing pressure, ocean acidification, habitat loss, changes in predation, etc.) would likely increase the rate and scale of estimated declines, with variation between species. For instance, Lam *et al*. (2014) used the DBEM to project the impacts of changes in SST, oxygen content (represented by O_2_ concentration), pH (represented by H^+^ concentration), and other variables such as salinity and ocean currents on the growth and distribution of marine species in the Arctic, and corresponding impacts on commercial fisheries by 2050 [108]. The inclusion of ocean acidification in these climate change projections reduced landings by 11% relative to the ‘climate change only’ scenario, with a corresponding 15% reduction in revenue in the Arctic by 2050 [108]. These projections have particular relevance for the species included in the present study, many of which are vulnerable to ocean acidification (e.g., Pacific halibut, sole, and invertebrates)[108], and suggest that impacts to food and economic security are likely to be more severe than those projected in this study. Ocean acidification has also been implicated in changes in competitive interactions between species, leading to ecosystem phase shifts [109]. Moreover, as this study does not account for all stages within the life cycles of diadromous species such as salmon, sturgeon, and eulachon, anthropogenic or environmental impacts on freshwater systems may also alter the projected results of this study for these species.

This study assumes that species’ current distributions are in equilibrium with environmental conditions [110,111], and thereby accurately reflect preferred habitat and environmental tolerance limits. However, given anthropogenic disturbances, it has been argued that ranges used in SDMs do not fully capture species’ historical ranges, which could lead to biases in estimates of species’ current environmental tolerance limits [84]. Within BC’s marine environment, such biases are likely less prominent as the majority of species included in this analysis have maintained their approximate historical range or occupy other areas with similar habitat, despite variations in abundance. Exceptions to this include invasive species, such as manila and varnish clams, and individual stocks of salmon and eulachon that have been extirpated.

Lastly, the use of inputs from different GCMs can yield a key source of uncertainty for DBEMs due to the direct relationship between the initial climate simulation and the estimates derived [112]. To incorporate this uncertainty, multi-model ensembles have been shown to improve projections by accounting for model-selection uncertainty and providing an ensemble of data that tends to agree better with observations [113]. Since this analysis evaluates scenarios generated from one coupled atmosphere-ocean global climate model (GFDL’s ESM2M), the inclusion of additional SDM-GCM combinations would help to further elucidate areas of uncertainty in model outputs, and to explore inter-model variation [36]. The resolution of current GCMs is most suited to large-scale analyses due to the intricacies of representing fine-scale ocean dynamics. In particular, there has been a concerted effort towards resolving coastal mixing processes and mesoscale eddies within GCMs [114]. While analyses at the scale of the EEZ can provide greater certainty with respect to regional impacts on fisheries, our understanding of impacts on coastal communities is therefore less certain due to both the complicated dynamics of coastal systems and the finer scale of the analysis.

#### Species’ phenological and genetic responses to environmental change

Despite globally consistent examples of poleward range shifts exhibited by species in response to climate change [2,4], recent studies have argued that focusing predominantly on unidirectional shifts may, in fact, underestimate the true impacts of climate change on species’ distributions due to the complexity of potential configurations of the variables that instigate the shifts [115]. As climatic niches are not necessarily associated with a latitudinally-defined gradient, habitat situated nearby, or closer to the equator, may provide refuge for species seeking new climate niches. While likely having little effect on projections at the global scale, finer-scale analyses such as this must acknowledge this uncertainty surrounding projections [115]. Likewise, while some pelagic species have exhibited much faster rates of poleward movement [116], rates of range expansion are dependent on the contrast of local temperature gradients, with strong variations in ‘climate velocities,’ or the rates and directions that isotherms shift through space [117,118].

For example, another study used a multi-model ensemble, including the DBEM, to project poleward range shifts of 26 to 28 km decade^-1^ in the UK’s waters [84], which are greater than the global median projected rates of 15.5 to 25.6 km decade^-1^ obtained using the same ensemble [42]. Furthermore, although demersal species were able to expand their ranges to deeper water if environmental conditions became more suitable within the model, pelagic species’ locations within the water column were not analysed in this study, but have been shown or projected to occur in response to climate change [70,119]. Since this study focused on species currently harvested by First Nations for economic and subsistence purposes, changes in the relative abundances and distributions of southern species that are likely to shift polewards into BC’s waters were not taken into account, and may offer new harvest opportunities for First Nations’ fisheries. However, the potential for these invasive species to replace the full range of ecosystem services and cultural significance derived from species traditionally harvested would require further scientific analysis and community-based research.

The rate at which different species can genetically and phenotypically evolve to accommodate environmental change is also an important area of uncertainty that has been explored through various studies [83,120,121]. To date, scope for genetic adaptation to meet the observed rate of environmental change has only been studied for a few species through laboratory experiments [122]. If adaptation to climate change were possible for marine species, our projections may represent an over-estimation of declines in catch potential. However, given the observed widespread responses of species to ocean temperature changes through shifts in distribution and phenology, long-term adaptation of marine species to continued environmental change may be unlikely.

## Conclusion

To the authors’ knowledge, this study offers the first attempt to develop quantitative projections of climate-related impacts on small-scale fisheries and to explore how traditional fisheries management strategies could be used to respond to specific challenges inferred from these projections. This scenario-based framework thereby provides an opportunity to proactively identify challenges that are likely to arise from climate change, with specific application to local risk assessments for small-scale, traditional fisheries.

The projected declines in relative catch potential across each of the coastal First Nations’ territories under both the lower (RCP 2.6) and upper (RCP 8.5) scenarios of climate change yield three key questions that are relevant to fisheries globally under climate change: (1) how do we mitigate impacts to fisheries catch potential that are unequally distributed? (2) how do we account for the externalization of impacts to fisheries? and (3) how can we effectively implement joint-management frameworks that balance objectives? While this study presents examples of potential climate-resilient pathways that might be used to respond to these challenges in the context of coastal British Columbia, including resource sharing agreements that redistribute impacts, local mariculture operations that contribute to offsetting declines in catch potential, and joint-management frameworks that facilitate collaborative management approaches, community-based research would be an essential requirement to identify viable options that fully accommodate each community’s respective concerns and values. Findings from this study provide the foundation to guide future research to address this set of questions. While the estimates derived from this analysis are not intended to represent fisheries management guidelines, the underlying trends that emerge illustrate the value of using quantitatively derived scenarios to examine potential site-specific impacts. For instance, the latitudinal trends in declining catch potential projected in this analysis can be used to develop management strategies for addressing spatial trade-offs associated with the redistribution of marine resources. Moreover, this approach suggests key stakeholders or species that might be affected, allowing for options to be discussed proactively.

## Supporting Information

S1 Methods Domestic fishing areas of BC First Nations included in analysis. Detailed methodology for deriving domestic fishing areas from Statement of Intent (SOI) boundaries submitted during the BC Treaty Process, with metadata and link to data source.
(PDF)

S1 Text Correlations between (A) cumulative change in relative catch potential and latitude, and (B) change in species’ catch potential (%) and latitude. Materials include cumulative change in relative catch potential by domestic fishing area (Table A) and number of species (*n*) whose catch potential (%) is projected to increase, decrease, or remain neutral within First Nations’ respective domestic fishing areas (DFAs)(Table B). Projections represent the lower (RCP 2.6) and upper (RCP 8.5) scenarios of climate change.
(PDF)

S2 Text Sensitivity analyses using a multi-model ensemble of projected changes in relative catch potential by species (Table A) and a multi-model ensemble of projected latitudinal range shifts by species (Table B). Results are ordered by least to greatest standard deviation. Projections from AquaMaps and Maxent obtained from Jones and Cheung (2014).
(PDF)

S1 Table Sample of 98 species included in the analysis, ordered alphabetically by common name. (PDF)

S2 Table Sampled commercially-caught species aggregated by fishery. The proportion of species included in the analysis relative to those included in each fishery’s quota is noted.
(XLSX)

S3 Table First Nations’ participation in British Columbia’s commercial fisheries by percentage and number held of available licenses. Not all licenses may be active. Detailed data outlining species used to calculate aggregated impacts to each commercial fishery are available by request.
(PDF)

S4 Table Projected change in relative abundance for 98 species under the lower (RCP 2.6) and upper (RCP 8.5) scenarios of climate change. Projections obtained using the Dynamic Bioclimate Envelope Model (DBEM).
(PDF)

S5 Table Projected latitudinal range shifts for 98 species by 2050 relative to 2000 under the lower (RCP 2.6) and upper (RCP 8.5) scenarios of climate change, derived from the Dynamic Bioclimate Envelope Model (DBEM). Ordered from greatest to least latitudinal range shift under RCP 8.5.
(PDF)

S6 Table Projected change in relative catch potential for 98 species under the lower (RCP 2.6) and upper (RCP 8.5) scenarios of climate change. Estimates obtained using projected changes in relative abundance and Eq 5. Values in red indicate an insignificant change in catch potential, with disagreement regarding the directionality of the projected change in catch.
(PDF)

S7 Table Sample of First Nations’ traditional fisheries management approaches and analogous Western fisheries management strategies. (PDF)

S1 Data Species distribution and life history data used in the analysis. This Access database includes the data used to run the DBEM.
(ACCDB)
