# Supplementary material for: Projected Scenarios for Coastal First Nations’ Fisheries Catch Potential under Climate Change: Management Challenges and Opportunities
Source: PLoS One. 2016 Jan 13;11(1):e0145285. doi: 10.1371/journal.pone.0145285 (PMC4711888; doi:10.1371/journal.pone.0145285)
Supplement: S1 Methods — Detailed methodology for deriving domestic fishing areas from Statement of Intent (SOI) boundaries submitted during the BC Treaty Process, with metadata and link to data source. (PDF) [file pone.0145285.s001.pdf]

DATA PROCESSING

First Nations' Domestic Fishing Areas

First Nations' domestic fishing areas (DFAs) were derived from the approximate Statement of Intent (SOI) boundaries registered with the BC Treaty Commission as of October 2004, which were converted to 0.5° latitudinal by 0.5° longitudinal grid-cells to correspond with global climate model grids. The scale of the grid-cells yielded over- and under-representation of DFA boundaries. For this reason, DFA polygons were converted to raster format in *ArcGIS* (version 10.1) using the 'maximum combined area' method, thereby ensuring that potential domestic fishing grounds were fully accounted for and that estimates represented the maximum future catch potential under climate change. Cells shared by more than one First Nation are denoted, but do not necessarily reflect an overlap in territorial claims due to the scale of the grid. While these boundaries do not signify the full extent of territory previously used by First Nations, particularly with respect to the sharing of resources between communities [1-4], the boundaries serve to illustrate approximate areas requested by First Nations for FSC fishing purposes at the finest scale that could be reconciled with global climate modelling (GCM) approaches.

References

1. Boas F (2002) Indian myths and legends from the North Pacific Coast of North America. Bouchard R, Kennedy D, editors. Vancouver: Talonbooks.
2. Trosper RL (2003) Resilience in pre-contact Pacific Northwest social ecological systems. Conservation Ecology 7(3): 6. URL: http://www.consecol.org/vol7/iss3/art6/
3. Haggan N, Turner NJ, Carpenter J, Jones JT, Mackie Q, et al. (2004) 12,000 years of change: linking traditional and modern ecosystem science in the Pacific Northwest. Victoria, BC. pp. 1–16. URL: http://www.academia.edu/download/30891940/12000yrs_-_Haggan_et_al.pdf.
4. Turner NJ, Jones JT (2000) Occupying the land: Traditional patterns of land and resource ownership among First Peoples of British Columbia. Conference paper presented at '*Constituting the Commons: Crafting Sustainable Commons in the New Millennium, the Eighth Biennial Conference of the International Association for the Study of Common Property*,' Bloomington, IN, May 31st to June 4th. 30 pp. URI: http://hdl.handle.net/10535/1952.

METADATA

Statement of Intent Boundary (ILRR)

The obtained shapefile contains all Statement of Intent (SOI) Boundaries registered with the BC Treaty Commission as of October 2004. The lines on the map represent the approximate boundaries of traditional territories described in First Nations Statements of Intent to negotiate treaties which have been submitted to, and accepted by, the B.C. Treaty Commission. They are illustrative only. Publication of this map does not imply that the First Nations, the Province of British Columbia, or the Government of Canada have agreed to the boundaries shown. SOI Boundaries should not be displayed at scales larger than the source scale. As these boundaries are approximate, the First Nations themselves should be contacted for the definitive locations of their boundaries. Only the First Nations name associated with the boundary is maintained as an attribute.

The dataset was produced by PWGSC for INAC under the Information Sharing Protocol for the Federal Treaty Negotiation Office, Indian and Northern Affairs Canada, and Business Solutions Branch, Ministry of Sustainable Resource Management for the BC Treaty Negotiations Office. The full dataset and associated metadata can be obtained from the following link.

https://apps.gov.bc.ca/pub/geometadata/metadataDetail.do?recordUID=36744&recordSet=ISO19115
