# Supplementary material for: Projected Scenarios for Coastal First Nations’ Fisheries Catch Potential under Climate Change: Management Challenges and Opportunities
Source: PLoS One. 2016 Jan 13;11(1):e0145285. doi: 10.1371/journal.pone.0145285 (PMC4711888; doi:10.1371/journal.pone.0145285)
Supplement: S1 Text — Materials include cumulative change in relative catch potential by domestic fishing area (Table A) and number of species (n) whose catch potential (%) is projected to increase, decrease, or remain neutral within First Nations’ respective domestic fishing areas (DFAs)(Table B). Projections represent the lower (RCP 2.6) and upper (RCP 8.5) scenarios of climate change. (PDF) [file pone.0145285.s002.pdf]

S1 Text. Correlations between (A) cumulative change in relative catch potential and latitude, and (B) change in species' catch potential (%) and latitude.

Table A. Cumulative change in relative catch potential by domestic fishing area, as projected under the lower (RCP 2.6) and upper (RCP 8.5) climate change scenarios. Average latitudes of domestic fishing areas were derived from 'Statement of Intent' boundaries.

DOMESTIC FISHING AREA	REGION	APPROXIMATE LATITUDE	RCP2.6 (%)	RCP8.5 (%)
Tsimshian[1]	North Coast	52° - 55°N	-4.6	-3.2
Haida	Haida Gwaii	51° - 55° N	-5.8	-6.6
Heiltsuk	Central Coast	51° - 53.5° N	-6.6	-7.9
'Namgis	Central Coast	50.5° - 51.0° N	-7.9	-8.2
Tla'amin	Strait of Georgia	49.0° - 50.5° N	-21.1	-22.0
Tsawwassen	Strait of Georgia	48.5° - 49.5° N	-15.2	-27.4
Maa-nulth	West Coast Vancouver Island	47.5° - 50.5° N	-25.8	-27.8
**EEZ**	**All**	**47.5° - 55° N**	**-4.5**	**-10.7**

[1] It is important to note that the territories represented in this analysis only includes those outlined in the Statements of Intent submitted during the BC Treaty Process. As such, not all members or territories within a Nation or group may be represented. For example, as of 2013, only five Nations of the Tsimshian peoples were members of the Tsimshian First Nations Treaty Society, which submitted the SOI map used in this analysis: Gitga'at, Kitasoo/Xaixais, Kitselas, Kitsumkalum, and Metlakatla (AANDC 2014). The Gitxa'ala Nation and Lax Kw'alaams were negotiating separately at that time.

Table B. Number of species (*n*) whose catch potential (%) is projected to increase, decrease, or remain neutral within First Nations' respective domestic fishing areas (DFAs) under both the lower (RCP 2.6) and upper (RCP 8.5) scenarios of climate change. DFAs are ordered latitudinally from north to south.

DOMESTIC FISHING AREA (# of species)	APPROX. MEAN LATITUDE	ABSOLUTE NUMBER OF SPECIES (PER CENT OF TOTAL NUMBER OF SPECIES)		
		Declining	Neutral (-/+)	Increasing
Tsimshian ($n = 96$)	53.5°N	**74** (77.1%)	**9** (9.4%)	**13** (13.5%)
Haida ($n = 96$)	52.9°N	**79** (82.3%)	**9** (9.4%)	**8** (8.3%)
Heiltsuk ($n = 96$)	52.2°N	**78** (81.3%)	**13** (13.5%)	**5** (5.2%)
'Namgis ($n = 91$)	50.8°N	**86 (**94.5%)	**2** (2.2%)	**3** (3.3%)
Tla'amin ($n = 91$)	49.7°N	**89** (97.8%)	**0** (0%)	**2** (2.2%)
Maa-nulth ($n = 98$)	49.1°N	**97** (99.0%)	**0** (0%)	**1** (1.0%)
Tsawwassen ($n = 95$)	49.05°N	**93** (97.9%)	**0** (0%)	**2** (2.1%)
EEZ ($n = 98$)	47.5 – 55.0°N	**84** (85.7%)	**11** (11.2%)	**3** (3.1%)
