# Supplementary material for: Projected Scenarios for Coastal First Nations’ Fisheries Catch Potential under Climate Change: Management Challenges and Opportunities
Source: PLoS One. 2016 Jan 13;11(1):e0145285. doi: 10.1371/journal.pone.0145285 (PMC4711888; doi:10.1371/journal.pone.0145285)
Supplement: S2 Text — Results are ordered by least to greatest standard deviation. Projections from AquaMaps and Maxent obtained from Jones and Cheung (2014). (PDF) [file pone.0145285.s003.pdf]

S2 Text. Sensitivity Analysis.

Table A. Multi-model ensemble of projected changes in relative catch potential by species. Results ordered by least to greatest standard deviation. Projections from AquaMaps and Maxent obtained from Jones and Cheung (2014; **[1]**).

Species	Common name	Change in relative catch potential (%)			Median	Std. dev. (σ)
		DBEM	AquaMaps [1]	Maxent [1]		
C. productus	Crab, red rock	-0.5	-3.3	0.0	-0.5	1.8
S. flavidus	Rockfish, yellowtail	-9.7	-9.3	-6.4	-9.3	1.8
O. kisutch	Salmon, coho	-15.1	-18.8	-16.1	-16.1	1.9
A. fimbria	Sablefish	-9.2	-5.1	-1.6	-5.1	3.8
P. staminea	Clam, Pacific littleneck	-8.9	-3.6	-0.3	-3.6	4.3
S. giganteus	Clam, butter	-5.0	-14.4	-7.1	-7.1	4.9
H. stenolepis	Halibut, Pacific	-13.2	-19.9	-10.0	-13.2	5.1
M. magister	Crab, Dungeness	-11.4	-6.3	-1.1	-6.3	5.2
O. mykiss	Steelhead	-7.6	-0.7	3.0	-0.7	5.4
O. keta	Salmon, chum	-11.8	-21.4	-21.1	-21.1	5.5
H. elassodon	Sole, flathead	-26.1	-33.5	-20.2	-26.1	6.7
L. bilineata	Sole, Rock	-28.4	-22.5	-14.5	-22.5	7.0
M. proximus	Tomcod, Pacific	-16.6	-6.8	-2.9	-6.8	7.1
A. stomias	Flounder, arrowtooh	-32.0	-23.4	-16.6	-23.4	7.7
O. elongatus	Cod, ling	-6.6	-22.2	-7.4	-7.4	8.8
P. abrupta	Geoduck, Pacific	5.1	N/A	-8.9	-1.9	9.9
C. nuttallii	Cockle, nuttall	-10.9	5.0	7.9	5.0	10.1
P. caurinus	Scallop, weathervane	-21.2	-0.4	-4.8	-4.8	11.0
O. lurida	Oyster, Olympia	-18.0	N/A	-1.4	-9.7	11.7
P. borealis	Shrimp, northern	-22.1	-4.2	0.5	-4.2	11.9
G. macrocephalus	Cod, Pacific	-35.4	-13.6	-12.8	-13.6	12.8
P. vetula	Sole, English	-28.4	-5.4	-3.5	-5.4	13.9
O. gorbuscha	Salmon, pink	-43.1	-11.6	-22.6	-22.6	16.0
C. gigas	Oyster, Pacific cupped	-18.5	8.0	11.2	8.0	16.3
P. goniurus	Shrimp, humpy	-37.6	-35.3	-6.8	-35.3	17.2
T. pacificus	Eulachon	-37.1	-5.0	-6.8	-6.8	18.0
S. sagax	Sardine, Pacific	44.4	4.8	6.3	6.3	22.4
C. pallasiii	Herring, Pacific	-49.2	-17.0	-2.2	-17.0	24.0
L. aspera	Sole, yellowfin	-26.0	-70.1	-26.6	-26.6	25.3
O. tshawystcha	Salmon, Chinook	-44.3	-68.2	-9.8	-44.3	29.4
P. hypsinotus	Shrimp, humpback	-83.1	-21.3	-10.3	-21.3	39.2
S. patula	Clam, Pacific razor	-13.8	64.3	-3.6	-3.6	42.5

[1] Jones MC, Cheung WWL (2014) Multi-model ensemble projections of climate change effects on global marine biodiversity. ICES Journal of Marine Science: fsu172. doi:10.1093/icesjms/fsu172.

Table B. Multi-model ensemble of projected latitudinal range shifts by species. Results ordered by least to greatest standard deviation. Projections from AquaMaps and Maxent obtained from Jones and Cheung (2014; **[1]**).

Species	Common name	Latitudinal range shift (km decade^{-1})			Median	Std. dev. (σ)
		DBEM	AquaMaps [1]	Maxent [1]		
M. proximus	Tomcod, Pacific	17.0	19.6	23.5	19.6	3.3
S. flavidus	Rockfish, yellowtail	23.7	22.2	30.0	23.7	4.1
P. staminea	Clam, Pacific littleneck	32.1	32.7	25.2	32.1	4.2
G. macrocephalus	Cod, Pacific	16.1	14.7	27.5	16.1	7.0
O. elongatus	Cod, ling	22.1	36.8	32.0	32.0	7.5
T. pacificus	Eulachon	39.5	24.8	27.5	27.5	7.8
P. caurinus	Scallop, weathervane	17.9	31.8	17.4	17.9	8.2
H. elassodon	Sole, flathead	13.3	8.9	25.5	13.3	8.6
A. fimbria	Sablefish	5.3	16.8	26.2	16.8	10.5
S. giganteus	Clam, butter	13.4	33.7	17.6	17.6	10.7
C. gigas	Oyster, Pacific cupped	75.3	83.6	62.3	75.3	10.7
A. stomias	Flounder, arrowtooth	17.3	2.6	23.6	17.3	10.8
M. magister	Crab, Dungeness	5.4	19.1	26.7	19.1	10.8
C. productus	Crab, red rock	19.3	41.1	26.8	26.8	11.1
L. bilineata	Sole, rock	9.5	17.0	32.9	17.0	11.9
H. stenolepis	Halibut, Pacific	3.3	21.8	25.9	21.8	12.0
P. vetula	Sole, English	14.6	21.5	40.8	21.5	13.6
P. borealis	Shrimp, northern	8.2	38.5	30.5	30.5	15.7
L. aspera	Sole, yellowfin	15.6	38.8	48.0	38.8	16.7
S. patula	Clam, Pacific razor	21.5	52.2	25.6	25.6	16.7
P. goniurus	Shrimp, humpy	0.7	12.6	36.1	12.6	18.0
P. hypsinotus	Shrimp, humpback	4.2	38.7	33.1	33.1	18.5
C. pallasii pallasii	Herring, Pacific	27.0	51.6	64.0	51.6	18.8
O. tshawytscha	Salmon, Chinook	27.4	5.8	47.9	27.4	21.1
O. keta	Salmon, chum	13.0	52.9	46.4	46.4	21.4
O. gorbuscha	Salmon, pink	23.6	71.2	40.6	40.6	24.1
O. mykiss	Steelhead	98.6	50.3	71.9	71.9	24.2
O. kisutch	Salmon, coho	7.7	21.9	58.9	21.9	26.4
P. abrupta	Geoduck, Pacific	13.8	NA	51.3	32.6	26.5
T. alalunga	Tuna, albacore	59.6	125.2	71.7	71.7	34.9
S. sagax	Sardine, Pacific	5.6	83.3	66.7	66.7	40.9
O. lurida	Oyster, Olympia	1.2	NA	79.4	40.3	55.3
C. nuttallii	Cockle, nuttall	21.7	33.3	129.9	33.3	59.4

[1] Jones MC, Cheung WWL (2014) Multi-model ensemble projections of climate change effects on global marine biodiversity. ICES Journal of Marine Science: fsu172. doi:10.1093/icesjms/fsu172.
