# Supplementary material for: Projected Scenarios for Coastal First Nations’ Fisheries Catch Potential under Climate Change: Management Challenges and Opportunities
Source: PLoS One. 2016 Jan 13;11(1):e0145285. doi: 10.1371/journal.pone.0145285 (PMC4711888; doi:10.1371/journal.pone.0145285)
Supplement: S1 Table — (PDF) [file pone.0145285.s004.pdf]

S1 Table. Sample of 98 species, ordered alphabetically by common name.

COMMON NAME	SCIENTIFIC NAME	DEMERSAL OR PELAGIC	FUNCTIONAL GROUP
Abalone, northern	*Haliotis kamtschatkana*	Demersal	Abalone
Barnacle, acorn	*Balanus glandula*	Demersal	Barnacle
Barnacle, giant acorn	*Balanus nubilus*	Demersal	Barnacle
Barnacle, gooseneck	*Pollicipes polymerus*	Demersal	Barnacle
Barnacle, thatched	*Semibalanus cariosus*	Demersal	Barnacle
Cabezon	*Scorpaenichthys marmoratus*	Demersal	Sculpin (Cottidae)
Chiton, black	*Katharina tunicata*	Demersal	Chiton
Chiton, giant Pacific	*Cryptochiton stelleri*	Demersal	Chiton
Clam, butter	*Saxidomus giganteus*	Demersal	Clam
Clam, horse	*Tresus capax*	Demersal	Clam
Clam, manila	*Venerupis philippinarum*	Demersal	Clam
Clam, Pacific littleneck	*Protothaca staminea*	Demersal	Clam
Clam, Pacific razor	*Siliqua patula*	Demersal	Clam
Clam, varnish	*Nuttallia obscurata*	Demersal	Clam
Cockle, nuttall	*Clinocardium nuttallii*	Demersal	Clam
Cod, Ling	*Ophiodon elongatus*	Demersal	Cod
Cod, Pacific	*Gadus macrocephalus*	Demersal	Cod
Crab, Dungeness	*Metacarcinus magister*	Demersal	Crab
Crab, green shore	*Hemigrapsus oregonensis*	Demersal	Crab
Crab, purple shore	*Hemigrapsus nudus*	Demersal	Crab
Crab, red rock	*Cancer productus*	Demersal	Crab
Crab, tanner	*Chionoecetes bairdi*	Demersal	Crab
Dogfish, spiny	*Squalus suckleyi*	Demersal	Elasmobranch
Eulachon	*Thaleichthys pacificus*	Pelagic	Eulachon
Flounder, arrowtooth	*Atheresthes stomias*	Demersal	Flounder and soles
Flounder, starry	*Platichthys stellatus*	Demersal	Flounder and soles
Gaper, Pacific	*Tresus nuttallii*	Demersal	Clam
Geoduck, Pacific	*Panopea abrupta*	Demersal	Geoduck
Greenling, kelp	*Hexagrammos decagrammus*	Demersal	Greenling (Hexagrammidae)
Halibut, Pacific	*Hippoglossus stenolepis*	Demersal	Halibut
Herring, Pacific	*Clupea pallasii pallasii*	Pelagic	Herring
Lamprey, Pacific	*Entosphenus tridentatus*	Demersal	Lamprey
Mussel, northern horse	*Modiolus modiolus*	Demersal	Mussel
Mussel, Pacific blue	*Mytilus trossulus*	Demersal	Mussel
Oyster, Olympia	*Ostrea lurida*	Demersal	Oyster
Oyster, Pacific cupped	*Crassostrea gigas*	Demersal	Oyster
Perch, kelp	*Brachyistius frenatus*	Demersal	Perch
Perch, pile	*Rhacochilus vacca*	Demersal	Perch
Red Irish lord	*Hemilepidotus hemilepidotus*	Demersal	Sculpin (Cottidae)
Rockfish, black	*Sebastes melanops*	Demersal	Rockfish (Scorpaenidae)
Rockfish, bocaccio	*Sebastes paucispinis*	Demersal	Rockfish (Scorpaenidae)
Rockfish, canary	*Sebastes pinniger*	Demersal	Rockfish (Scorpaenidae)
Rockfish, chilipepper	*Sebastes goodei*	Demersal	Rockfish (Scorpaenidae)

COMMON NAME	SCIENTIFIC NAME	DEMERSAL OR PELAGIC	FUNCTIONAL GROUP
Rockfish, China	*Sebastes nebulosus*	Demersal	Rockfish (Scorpaenidae)
Rockfish, copper	*Sebastes caurinus*	Demersal	Rockfish (Scorpaenidae)
Rockfish, dusky	*Sebastes ciliatus*	Demersal	Rockfish (Scorpaenidae)
Rockfish, quillback	*Sebastes maliger*	Demersal	Rockfish (Scorpaenidae)
Rockfish, redbanded	*Sebastes babcocki*	Demersal	Rockfish (Scorpaenidae)
Rockfish, redstripe	*Sebastes proriger*	Demersal	Rockfish (Scorpaenidae)
Rockfish, rougheye	*Sebastes aleutianus*	Demersal	Rockfish (Scorpaenidae)
Rockfish, shortraker	*Sebastes borealis*	Demersal	Rockfish (Scorpaenidae)
Rockfish, silvergray	*Sebastes brevispinis*	Demersal	Rockfish (Scorpaenidae)
Rockfish, tiger	*Sebastes nigrocinctus*	Demersal	Rockfish (Scorpaenidae)
Rockfish, vermillion	*Sebastes miniatus*	Demersal	Rockfish (Scorpaenidae)
Rockfish, widow	*Sebastes entomelas*	Demersal	Rockfish (Scorpaenidae)
Rockfish, yelloweye	*Sebastes ruberrimus*	Demersal	Rockfish (Scorpaenidae)
Rockfish, yellowmouth	*Sebastes reedi*	Demersal	Rockfish (Scorpaenidae)
Rockfish, yellowtail	*Sebastes flavidus*	Demersal	Rockfish (Scorpaenidae)
Sablefish	*Anoplopoma fimbria*	Demersal	Sablefish
Salmon, chinook	*Oncorhynchus tshawytscha*	Demersal	Salmon
Salmon, chum	*Oncorhynchus keta*	Demersal	Salmon
Salmon, coho	*Oncorhynchus kisutch*	Demersal	Salmon
Salmon, pink	*Oncorhynchus gorbuscha*	Demersal	Salmon
Salmon, sockeye	*Oncorhynchus nerka*	Demersal	Salmon
Sanddab, Pacific	*Citharichthys sordidus*	Demersal	Flounder and soles
Sardine, Pacific	*Sardinops sagax*	Pelagic	Sardine
Scallop, rock	*Crassadoma gigantea*	Demersal	Scallop
Scallop, spiny	*Chlamys hastata*	Demersal	Scallop
Scallop, weathervane	*Patinopecten caurinus*	Demersal	Scallop
Sculpin, dusky	*Icelinus burchami*	Demersal	Sculpin (Cottidae)
Sculpin, longfin	*Jordania zonope*	Demersal	Sculpin (Cottidae)
Sculpin, Puget Sound	*Ruscarius meanyi*	Demersal	Sculpin (Cottidae)
Sculpin, spotfin	*Icelinus tenuis*	Demersal	Sculpin (Cottidae)
Sculpin, thornback	*Paricelinus hopliticus*	Demersal	Sculpin (Cottidae)
Sea cucumber, red	*Parastichopus californicus*	Demersal	Echinoderms
Sea urchin, green	*Strongylocentrotus droebachiensis*	Demersal	Echinoderms
Sea urchin, red	*Mesocentrotus franciscanus*	Demersal	Echinoderms
Shrimp, coonstripe	*Pandalus danae*	Demersal	Prawns and shrimp
Shrimp, humpback	*Pandalus hypsinotus*	Demersal	Prawns and shrimp
Shrimp, humpy	*Pandalus goniurus*	Demersal	Prawns and shrimp
Shrimp, northern	*Pandalus borealis*	Demersal	Prawns and shrimp
Shrimp, spot	*Pandalus platyceros*	Demersal	Prawns and shrimp
Skate, longnose	*Raja rhina*	Demersal	Elasmobranch
Sole, English	*Parophrys vetula*	Demersal	Flounder and soles
Sole, flathead	*Hippoglossoides elassodon*	Demersal	Flounder and soles
Sole, Pacific dover	*Microstomus pacificus*	Demersal	Flounder and soles
Sole, petrale	*Eopsetta jordani*	Demersal	Flounder and soles

**COMMON NAME**	**SCIENTIFIC NAME**	**DEMERSAL OR PELAGIC**	**FUNCTIONAL GROUP**
Sole, rex	*Glyptocephalus zachirus*	Demersal	Flounder and soles
Sole, rock	*Lepidopsetta bilineata*	Demersal	Flounder and soles
Sole, yellowfin	*Limanda aspera*	Demersal	Flounder and soles
Steelhead	*Oncorhynchus mykiss*	Demersal	Trout
Sturgeon, white	*Acipenser transmontanus*	*Demersal*	Sturgeon
Thornyhead, rockfish	*Sebastolobus altivelis*	Demersal	Rockfish (Scorpaenidae)
Thornyhead, shortspine	*Sebastolobus alascanus*	Demersal	Rockfish (Scorpaenidae)
Tomcod, Pacific	*Microgadus proximus*	Demersal	Cod
Trout, cutthroat	*Oncorhynchus clarkii clarkii*	Demersal	Trout
Trout, Dolly Varden	*Salvelinus malma malma*	Demersal	Trout
Tuna, albacore	*Thunnus alalunga*	Pelagic	Tuna
