# Supplementary material for: Projected Scenarios for Coastal First Nations’ Fisheries Catch Potential under Climate Change: Management Challenges and Opportunities
Source: PLoS One. 2016 Jan 13;11(1):e0145285. doi: 10.1371/journal.pone.0145285 (PMC4711888; doi:10.1371/journal.pone.0145285)
Supplement: S3 Table — Not all licenses may be active. Detailed data outlining species used to calculate aggregated impacts to each commercial fishery are available by request. (PDF) [file pone.0145285.s006.pdf]

S3 Table. First Nations' participation in British Columbia's commercial fisheries by percentage and number of total licenses held (modified from **[1]** and **[2]**)[1,8].

Commercial License	Common Name(s)	Scientific Name(s)	Per Cent and Number of Licenses Held (2003)
Haida razor clam	Pacific razor clam	*Siliqua patula*	100% (94 – 269[2])
Heiltsuk intertidal clam	Manila clam, Pacific littleneck clam	*Venerupis philippinarum, Protothaca staminea*	100% (50)
Herring spawn-on-kelp	Pacific herring	*Clupea pallasii pallasii*	78.3% (36)[3]
Sardine by seine	Pacific sardine	*Sardinops sagax*	58% (29)
Clam by hand	Butter clam, manila clam, Pacific littleneck clam	*Saxidomus gigantea, Venerupis philippinarum, Protothaca staminea*	56.5% (648)
Salmon seine (Areas A, B)	Salmon	*Oncorhynchus* spp.	29.0% (80)[4]
Salmon gillnet (Areas C, D, E)	Salmon	*Oncorhynchus* spp.	38.1% (536)
Roe herring seine	Pacific herring	*Clupea pallasii*	25.0% (63)[5]
Roe herring gillnet	Pacific herring	*Clupea pallasii*	27.5% (345)[5]
Red sea urchin	Red sea urchin	*Mesocentrotus franciscanus*	12.7% (14)
Eulachon[6]	Eulachon	*Thaleichthys pacificus*	12.5% (2)
Halibut	Pacific halibut	*Hippoglossus stenolepis*	12.2% (53)
Sea cucumber	Giant red sea cucumber	*Parastichopus californicus*	11.8% (10)
Salmon troll (Areas F, G, H)	Salmon	*Oncorhynchus* spp.	9.3% (50)
Rockfish by hook and line	Rockfish	*Sebastes* spp., *Sebastolobus* spp.	7.3% (19)
Shrimp trawl	Humpback, northern pink, pink, sidestripe	*Pandalus* spp. *Pandalopsis dispar*	6.1% (15)
Crab	Dungeness crab, Pacific rock crab	*Metacarcinus magister, Cancer productus*	5% (11)
Sablefish	Sablefish	*Anoplopoma fimbria*	4.2% (2)
Category C (hook and line)	Schedule II species[7]	--	3.7% (20)
Prawn	Prawn	*Pandalus platyceros*	3.6% (9)
Groundfish trawl (T)	Pacific sanddab, sculpins, greenlings, including Schedule II[7]	*Citharichthys sordidus, Cottidae* spp., *Hexagrammos* spp., etc.[7]	3.5% (5)

Commercial License	Common Name(s)	Scientific Name(s)	Per Cent and Number of Licenses Held (2003)
Geoduck and horse clam	Horse clam, Pacific geoduck, Pacific gaper	*Tresus capax, Panopea abrupta, Tresus nuttallii*	1.8% (1)

[1] Note that not all licenses may be active.
[2] As of 2003, an unlimited number of harvesters were allowed under the Haida Communal Clam Licence **[1]**.
[3] 80.8% of the quota was held in 2003 **[1]**.
[4] Inclusion of Aboriginal-operated licenses increases the percentage to 46.7% **[1]**.
[5] Percentage held of active licenses [1].
[6] Commercial fishing is no longer permitted due to conservation status.
[7] Outlined under *Schedule II – Part II* of Canada's *Pacific Fisheries Regulations (1993)*.
[8] Species used to calculate aggregated impacts to each commercial fishery are outlined in **S4 Table**.

References

1. James M (2003) Native Participation in British Columbia Commercial Fisheries – 2003. Ministry of Agriculture, Food and Fisheries. 35 pp. Accessed from: http://www.al.gov.bc.ca/fisheries/cabinet/NativeParticipationBCFishing03.pdf.
2. Heiltsuk First Nation (2011) Heiltsuk Integrated Marine Use Plan. 118 pp. Accessed from: http://bit.ly/1NdA78I.
