# Supplementary material for: Projected Scenarios for Coastal First Nations’ Fisheries Catch Potential under Climate Change: Management Challenges and Opportunities
Source: PLoS One. 2016 Jan 13;11(1):e0145285. doi: 10.1371/journal.pone.0145285 (PMC4711888; doi:10.1371/journal.pone.0145285)
Supplement: S4 Table — Projections obtained using the Dynamic Bioclimate Envelope Model (DBEM). (PDF) [file pone.0145285.s007.pdf]

S4 Table. Projected change in relative abundance for 98 species under the lower (RCP 2.6) and upper (RCP 8.5) scenarios of climate change. Projections obtained from the Dynamic Bioclimate Envelope Model (DBEM).

LEGEND	Decline	Increase

TAXON KEY	SPECIES	RCP 2.6 Difference (%)	RCP 8.5 Difference (%)
690617	Humpback shrimp	-75.0	-84.7
690615	Sidestriped shrimp	-45.0	-70.6
601520	Pacific herring	-31.8	-48.7
600244	Chinook salmon	-47.8	-46.8
600240	Pink salmon	-40.3	-44.1
604009	Shortspine thornyhead	-44.8	-41.9
604238	Rex sole	-39.3	-39.7
700022	Giant Pacific chiton	-18.9	-37.2
690616	Humpy shrimp	-18.2	-37.1
600243	Sockeye salmon	-10.2	-36.2
690494	Green sea urchin	-16.9	-36.0
600256	*Eulachon*	-26.4	-35.7
600308	Pacific cod	-12.6	-35.0
700034	Red Irish lord	-10.2	-34.4
700040	Northern horse mussel	-10.9	-33.2
600517	Arrowtooth flounder	-34.1	-32.0
604247	Pacific dover sole	-11.5	-31.3
700006	Redstripe rockfish	-9.5	-29.3
604249	Starry flounder	-11.2	-29.2
604215	Pacific sanddab	-10.5	-28.8
604248	English sole	-32.0	-28.6
624237	Rock sole	-12.4	-28.3
700033	Dolly Varden trout	-10.8	-28.1
604237	Petrale sole	-11.5	-27.1
700007	Longspine thornyhead	-12.4	-27.0
600520	Yellowfin sole	-8.0	-25.6
700044	Purple shore crab	-17.3	-25.5
600519	Flathead sole	-24.8	-24.8
600142	Albacore tuna	-20.2	-23.9
690269	Prawn / northern shrimp	-13.8	-22.1
700045	Green shore crab	-14.6	-21.9
690429	Weathervane scallop	-11.6	-21.2
700041	Black chiton	-14.2	-20.3
690283	Pacific cupped oyster	-23.8	-18.9
700010	Shortraker rockfish	-7.8	-17.8
690279	Olympia oyster	-11.8	-17.7
700055	Spiny dogfish	-10.0	-17.7
700011	Quillback rockfish	-8.6	-17.5

LEGEND	Decline	Increase

TAXON KEY	SPECIES	RCP 2.6 Difference (%)	RCP 8.5 Difference (%)
601879	Pacific tomcod	-22.1	-17.0
700026	Cutthroat trout	-9.4	-16.6
700043	Tanner crab	-17.4	-16.0
700023	Red sea urchin	-10.3	-15.7
600245	Coho salmon	-8.8	-15.2
700017	Redbanded rockfish	-8.4	-15.1
603989	Canary rockfish	-9.9	-15.0
600502	Widow rockfish	-11.0	-14.9
700001	Red sea cucumber	-12.8	-14.9
700029	Pacific blue mussel	-10.2	-14.6
700051	Spot shrimp/prawn	-11.0	-14.1
700047	Giant acorn barnacle	-13.6	-14.1
690286	Pacific razor clam	-13.8	-14.1
700019	*Northern abalone*	-10.5	-13.9
604140	Cabezon	-9.0	-13.4
700030	Longnose skate	-9.7	-13.3
700018	Dusky rockfish	-4.9	-13.2
600514	Pacific halibut	-12.3	-13.0
700042	Thatched barnacle	-13.2	-12.3
600241	Chum salmon	-9.6	-12.1
700037	Longfin sculpin	-8.5	-11.8
690115	Dungeness crab	-13.3	-11.6
700046	Acorn barnacle	-12.4	-11.5
700039	Spotfin sculpin	-8.7	-11.3
690651	Nuttall cockle	-12.6	-11.1
700009	Rougheye rockfish	-5.4	-10.8
700036	Puget Sound sculpin	-7.9	-10.8
603987	Bocaccio rockfish	-8.9	-10.5
700027	Horse clam	-13.6	-10.2
600503	Yellowtail rockfish	-8.8	-10.2
690664	Pacific gaper	-9.5	-9.9
700020	Varnish clam	-15.4	-9.8
700002	Silvergray rockfish	-8.4	-9.5
700054	Spiny scallop	-9.0	-9.4
600512	Sablefish	-10.8	-9.2
690285	Pacific littleneck clam	-8.3	-9.0
700035	Dusky sculpin	-8.2	-8.6
600239	Steelhead	-9.8	-8.0
600509	Lingcod	-8.7	-7.3
700012	Copper rockfish	-8.6	-7.0
603971	Chilipepper rockfish	-9.3	-6.3
700005	Yelloweye rockfish	-8.1	-6.3

LEGEND	Decline	Increase

TAXON KEY	SPECIES	RCP 2.6 Difference (%)	RCP 8.5 Difference (%)
690064	Butter clam	-9.3	-5.5
700052	Gooseneck barnacle	-7.5	-5.3
700003	Pacific lamprey	-8.1	-4.9
700031	Pile perch	-9.4	-3.4
700021	Rock scallop	-3.5	-2.9
690287	Red rock crab	-7.7	-1.5
700015	Tiger rockfish	-5.7	-0.7
700008	Yellowmouth rockfish	-6.1	0.3
700032	Kelp perch	-7.7	0.6
700013	China rockfish	-7.0	1.0
700014	Kelp greenling	-7.7	2.2
603979	Black rockfish	-6.5	3.1
700038	Thornback sculpin	-7.2	3.8
690284	Pacific geoduck	-8.3	4.1
700016	Vermillion rockfish	-8.0	4.6
700025	Manila clam	-1.3	14.5
602594	*White sturgeon*	0.5	18.4
700050	Pacific sardine	32.9	44.3
	**Total change in abundance (%)**	**-9.9**	**-10.1**
