# Supplementary material for: Projected Scenarios for Coastal First Nations’ Fisheries Catch Potential under Climate Change: Management Challenges and Opportunities
Source: PLoS One. 2016 Jan 13;11(1):e0145285. doi: 10.1371/journal.pone.0145285 (PMC4711888; doi:10.1371/journal.pone.0145285)
Supplement: S5 Table — Ordered from greatest to least latitudinal range shift under RCP 8.5. (PDF) [file pone.0145285.s008.pdf]

S5 Table. Projected range shifts for 98 species by 2050 relative to 2000 under the lower (RCP 2.6) and upper (RCP 8.5) scenarios of climate change, derived from the Dynamic Bioclimate Envelope Model (DBEM). Ordered from greatest to least latitudinal range shift under RCP 8.5.

Species	Common name	Demersal or pelagic	Latitudinal range shift (km decade^{-1})	
			RCP 2.6	RCP 8.5
Vermillion rockfish	Rockfish (Scorpaenidae)	Demersal	170.8	164.8
Steelhead	Steelhead	Demersal	117.9	98.6
Pacific cupped oyster	Oyster	Demersal	0.3	75.3
Sidestriped shrimp	Prawns and shrimp	Demersal	9.6	61.9
Green shore crab	Crab	Demersal	35.7	60.8
Albacore tuna	Tuna	Pelagic	1.3	59.6
Gooseneck barnacle	Barnacle	Demersal	32.6	54.0
Yelloweye rockfish	Rockfish (Scorpaenidae)	Demersal	24.8	51.0
Cabezon	Sculpin (Cottidae)	Demersal	0.0	43.8
Eulachon	Eulachon	Pelagic	32.9	39.5
Spotfin sculpin	Sculpin (Cottidae)	Demersal	0.8	39.0
Longnose skate	Elasmobranch	Demersal	22.2	38.2
White sturgeon	Sturgeon	Demersal	24.9	36.4
Kelp greenling	Greenling (Hexagrammidae)	Demersal	23.4	35.7
Purple shore crab	Crab	Demersal	35.0	34.3
Giant Pacific chiton	Chiton	Demersal	19.4	33.6
Northern horse mussel	Mussel	Demersal	12.6	33.5
Copper rockfish	Rockfish (Scorpaenidae)	Demersal	29.1	32.8
Chilipepper rockfish	Rockfish (Scorpaenidae)	Demersal	2.6	32.6
Pacific littleneck clam	Clam	Demersal	54.8	32.1
Redstripe rockfish	Rockfish (Scorpaenidae)	Demersal	19.3	30.6
Spiny scallop	Scallop	Demersal	29.0	27.5
Chinook salmon	Salmon	Demersal	24.6	27.4
Pacific herring	Herring	Pelagic	15.2	27.0
Widow rockfish	Rockfish (Scorpaenidae)	Demersal	13.9	26.5
Tanner crab	Crab	Demersal	14.5	25.3
Canary rockfish	Rockfish (Scorpaenidae)	Demersal	10.5	24.8
Yellowtail rockfish	Rockfish (Scorpaenidae)	Demersal	9.7	23.7
Quillback rockfish	Rockfish (Scorpaenidae)	Demersal	24.2	23.7
Pink salmon	Salmon	Demersal	16.7	23.6
Black rockfish	Rockfish (Scorpaenidae)	Demersal	9.9	23.4
Green sea urchin	Echinoderms	Demersal	15.4	23.2

Species	Common name	Demersal or pelagic	Latitudinal range shift (km decade^{-1})	
			RCP 2.6	RCP 8.5
Pacific lamprey	Lamprey	Demersal	10.1	22.7
Tiger rockfish	Rockfish (Scorpaenidae)	Demersal	13.9	22.6
Kelp perch	Perch	Demersal	8.6	22.5
Silvergray rockfish	Rockfish (Scorpaenidae)	Demersal	19.7	22.4
Dusky rockfish	Rockfish (Scorpaenidae)	Demersal	10.0	22.4
Lingcod	Lingcod	Demersal	9.7	22.1
Bocaccio rockfish	Rockfish (Scorpaenidae)	Demersal	9.6	21.7
Nuttall cockle	Clam	Demersal	9.6	21.7
Pacific razor clam	Clam	Demersal	26.3	21.5
Pile perch	Perch	Demersal	2.4	21.5
Black chiton	Chiton	Demersal	25.5	20.9
Petrale sole	Flounder and soles	Demersal	11.5	20.2
Starry flounder	Flounder and soles	Demersal	6.2	20.2
Thatched barnacle	Barnacle	Demersal	23.9	20.2
Red rock crab	Crab	Demersal	11.4	19.3
Giant acorn barnacle	Barnacle	Demersal	13.4	18.4
Pacific gaper	Clam	Demersal	13.6	18.1
Weathervane scallop	Scallop	Demersal	17.6	17.9
Puget Sound sculpin	Sculpin (Cottidae)	Demersal	7.3	17.7
Longfin sculpin	Sculpin (Cottidae)	Demersal	8.1	17.7
Arrowtooth flounder	Flounder and soles	Demersal	18.1	17.3
Rex sole	Flounder and soles	Demersal	14.5	17.3
Northern abalone	Abalone	Demersal	12.3	17.3
Pacific tomcod	Pacific cod	Demersal	13.6	17.0
Acorn barnacle	Barnacle	Demersal	19.4	16.7
Pacific cod	Pacific cod	Demersal	6.1	16.1
Thornback sculpin	Sculpin (Cottidae)	Demersal	4.6	16.1
Pacific sanddab	Flounder and soles	Demersal	13.6	15.9
Varnish clam	Clam	Demersal	9.3	15.9
Yellowfin sole	Flounder and soles	Demersal	6.0	15.6
Pacific dover sole	Flounder and soles	Demersal	11.2	15.2
English sole	Flounder and soles	Demersal	16.0	14.6
Pacific geoduck	Geoduck	Demersal	14.2	13.8
Spot shrimp/prawn	Prawns and shrimp	Demersal	15.3	13.7
Yellowmouth rockfish	Rockfish (Scorpaenidae)	Demersal	15.7	13.5

Species	Common name	Demersal or pelagic	Latitudinal range shift (km $decade^{-1}$)	
			RCP 2.6	RCP 8.5
Butter clam	Clam	Demersal	26.0	13.4
Flathead sole	Flounder and soles	Demersal	13.5	13.3
Chum salmon	Salmon	Demersal	7.0	13.0
Shortspine thornyhead	Rockfish (Scorpaenidae)	Demersal	9.5	11.6
Pacific blue mussel	Mussel	Demersal	7.6	11.5
Dolly Varden trout	Trout	Demersal	6.2	10.0
Cutthroat trout	Trout	Demersal	7.9	9.6
Rock sole	Flounder and soles	Demersal	1.9	9.5
Red Irish lord	Sculpin (Cottidae)	Demersal	3.3	9.2
Rock scallop	Scallop	Demersal	9.2	8.8
Prawn / northern shrimp	Prawns and shrimp	Demersal	2.2	8.2
Red sea urchin	Echinoderms	Demersal	6.5	7.9
Coho salmon	Salmon	Demersal	6.1	7.7
China rockfish	Rockfish (Scorpaenidae)	Demersal	7.1	7.4
Shortraker rockfish	Rockfish (Scorpaenidae)	Demersal	2.1	7.1
Sockeye salmon	Salmon	Demersal	4.2	6.5
Red sea cucumber	Echinoderms	Demersal	8.2	6.3
Manila clam	Clam	Demersal	1.1	6.3
Horse clam	Clam	Demersal	10.0	6.1
Pacific sardine	Sardine	Pelagic	12.0	5.6
Dungeness crab	Crab	Demersal	9.9	5.4
Sablefish	Sablefish	Demersal	6.8	5.3
Humpback shrimp	Prawns and shrimp	Demersal	26.5	4.2
Pacific halibut	Halibut	Demersal	1.0	3.3
Rougheye rockfish	Rockfish (Scorpaenidae)	Demersal	0.1	2.6
Redbanded rockfish	Rockfish (Scorpaenidae)	Demersal	4.7	2.5
Olympia oyster	Oyster	Demersal	3.4	1.2
Dusky sculpin	Sculpin (Cottidae)	Demersal	5.4	0.9
Humpy shrimp	Prawns and shrimp	Demersal	3.5	0.7
Spiny dogfish	Elasmobranch	Demersal	0.4	0.1
Longspine thornyhead	Rockfish (Scorpaenidae)	Demersal	3.1	0.0
		**Pelagic (median)**	**13.6**	**33.3**
		**Demersal (median)**	**10.1**	**17.8**
		**All (median)**	**10.3**	**18.0**
