# Supplementary material for: Projected Scenarios for Coastal First Nations’ Fisheries Catch Potential under Climate Change: Management Challenges and Opportunities
Source: PLoS One. 2016 Jan 13;11(1):e0145285. doi: 10.1371/journal.pone.0145285 (PMC4711888; doi:10.1371/journal.pone.0145285)
Supplement: S6 Table — Estimates obtained using projected changes in relative abundance and Eq 5. Values in red indicate an insignificant change in catch potential, with disagreement regarding the directionality of the projected change in catch. (PDF) [file pone.0145285.s009.pdf]

S6 Table. Projected change in relative catch potential for 98 species under the lower (RCP 2.6) and upper (RCP 8.5) scenarios of climate change by 2050 relative to 2000. Estimates obtained using projected changes in relative abundance and Equation 5. Values in red indicate an insignificant change in catch potential, with disagreement regarding the directionality of the projected change in catch.

Species	Common name	RCP 2.6		RCP 8.5		Change in catch potential (%)	
		2000	2050	2000	2050	RCP 2.6	RCP 8.5
Sardinops sagax	Pacific sardine	38,620,251	54,834,600	36,208,376	52,294,469	42.0	44.4
Acipenser transmontanus	White sturgeon	309,160,459	333,223,046	305,503,727	365,139,896	7.8	19.5
Venerupis philippinarum	Manila clam	286,428,532	302,808,911	284,631,152	328,462,256	5.7	15.4
Sebastes miniatus	Vermillion rockfish	988,622,229	971,564,242	984,935,643	1,039,220,601	-1.7	5.5
Panopea abrupta	Pacific geoduck	75,349,615	74,232,419	74,272,850	78,065,233	-1.5	5.1
Paricelinus hopliticus	Thornback sculpin	1,414,760,719	1,403,219,579	1,414,760,719	1,436,238,492	-0.8	4.6
Sebastes melanops	Black rockfish	285,585,167	285,349,111	279,824,815	290,590,029	-0.1	3.8
Hexagrammos decagrammus	Kelp greenling	112,921,120	111,480,439	109,262,370	112,148,943	-1.3	2.6
Sebastes nebulosus	China rockfish	1,154,153,650	1,146,141,547	1,129,409,322	1,148,807,715	-0.7	1.7
Brachyistius frenatus	Kelp perch	1,078,180,129	1,066,084,996	1,046,718,930	1,054,730,260	-1.1	0.8
Sebastes reedi	Yellowmouth rockfish	779,899,243	779,207,863	780,444,683	785,711,471	-0.1	0.7
Sebastes nigrocinctus	Tiger rockfish	659,762,955	663,208,941	657,103,577	654,163,877	0.5	-0.4
Cancer productus	Red rock crab	926,231,530	916,411,181	922,622,468	917,819,109	-1.1	-0.5
Rhacochilus vacca	Pile perch	1,228,692,287	1,188,685,837	1,198,488,961	1,162,981,084	-3.3	-3.0
Entosphenus tridentatus	Pacific lamprey	269,624,551	264,056,080	268,889,696	256,385,721	-2.1	-4.7
Pollicipes polymerus	Gooseneck barnacle	531,365,864	525,152,452	528,297,178	502,701,236	-1.2	-4.8
Saxidomus giganteus	Butter clam	440,415,541	427,112,809	434,148,952	412,371,922	-3.0	-5.0
Sebastes ruberrimus	Yelloweye rockfish	399,130,815	390,603,671	399,156,740	374,280,448	-2.1	-6.2
Ophiodon elongatus	Lingcod	373,737,539	364,973,195	369,189,827	344,941,498	-2.3	-6.6
Sebastes goodei	Chilipepper rockfish	523,683,456	507,563,961	516,690,227	482,755,030	-3.1	-6.6
Sebastes caurinus	Copper rockfish	683,734,778	666,757,823	683,102,561	636,272,241	-2.5	-6.9
Oncorhynchus mykiss	Steelhead	126,196,031	121,543,558	124,383,093	114,941,144	-3.7	-7.6
Icelinus burchami	Dusky sculpin	1,005,490,993	984,424,780	1,011,013,125	928,084,170	-2.1	-8.2
Protothaca staminea	Pacific littleneck clam	458,766,096	448,930,066	455,618,798	414,915,144	-2.1	-8.9

Species	Common name	RCP 2.6		RCP 8.5		Change in catch potential (%)	
		2000	2050	2000	2050	RCP 2.6	RCP 8.5
Anoplopoma fimbria	Sablefish	45,413,229	43,086,614	45,961,186	41,726,292	-5.1	-9.2
Chlamys hastata	Spiny scallop	497,686,961	483,825,060	494,501,668	448,865,916	-2.8	-9.2
Crassadoma gigantea	Rock scallop	3,660,265,764	3,530,868,625	3,667,473,264	3,317,058,785	-3.5	-9.6
Sebastes flavidus	Yellowtail rockfish	395,633,872	385,611,628	391,682,661	353,736,111	-2.5	-9.7
Tresus nuttallii	Pacific gaper	1,295,361,057	1,252,055,247	1,292,065,019	1,167,315,663	-3.3	-9.7
Sebastes brevispinis	Silvergray rockfish	193,294,859	188,639,496	193,838,687	175,072,992	-2.4	-9.7
Nuttallia obscurata	Varnish clam	121,586,915	109,661,772	118,552,926	107,064,709	-9.8	-9.7
Sebastes paucispinis	Boccaccio rockfish	246,501,238	239,878,016	244,417,690	219,958,591	-2.7	-10.0
Ruscarius meanyi	Puget Sound sculpin	823,294,385	810,631,709	806,364,881	725,532,127	-1.5	-10.0
Tresus capax	Horse clam	886,225,032	817,412,967	882,940,330	792,944,873	-7.8	-10.2
Clinocardium nuttallii	Nuttall cockle	52,077,886	48,755,484	52,506,803	46,784,029	-6.4	-10.9
Sebastes aleutianus	Rougeheye rockfish	131,461,311	132,197,546	132,115,526	117,400,556	0.6	-11.1
Metacarcinus magister	Dungeness crab	876,382,194	813,834,829	876,453,491	776,907,680	-7.1	-11.4
Balanus glandula	Acorn barnacle	105,759,894	98,771,461	104,485,096	92,585,055	-6.6	-11.4
Jordania zonope	Longfin sculpin	1,330,437,053	1,302,537,859	1,320,187,426	1,168,844,968	-2.1	-11.5
Oncorhynchus keta	Chum salmon	102,683,421	99,148,477	102,460,001	90,352,910	-3.4	-11.8
Icelinus tenuis	Spotfin sculpin	693,020,400	673,386,200	695,816,318	612,971,469	-2.8	-11.9
Semibalanus cariosus	Thatched barnacle	436,853,690	404,885,800	436,055,008	382,276,965	-7.3	-12.3
Hippoglossus stenolepis	Pacific halibut	25,424,332	23,634,023	25,604,456	22,218,054	-7.0	-13.2
Raja rhina	Longnose skate	361,317,457	348,135,605	360,591,772	312,514,894	-3.6	-13.3
Scorpaenichthys marmoratus	Cabezon	901,464,524	874,276,666	891,027,337	771,030,578	-3.0	-13.5
Siliqua patula	Pacific razor clam	421,547,934	388,434,366	419,872,614	361,865,169	-7.9	-13.8
Sebastes ciliatus	Dusky rockfish	662,390,997	668,553,026	658,686,465	567,233,181	0.9	-13.9
Haliotis kamtschatkana	Northern abalone	1,297,158,438	1,236,780,874	1,289,446,004	1,109,071,797	-4.7	-14.0
Balanus nubilus	Giant acorn barnacle	183,565,583	169,349,544	183,211,803	157,422,274	-7.7	-14.1
Pandalus platyceros	Spot shrimp/prawn	1,062,913,566	1,009,177,809	1,055,089,002	906,398,660	-5.1	-14.1
Sebastes entomelas	Widow rockfish	889,289,244	848,165,389	890,279,236	763,399,563	-4.6	-14.3

Species	Common name	RCP 2.6		RCP 8.5		Change in catch potential (%)	
		2000	2050	2000	2050	RCP 2.6	RCP 8.5
Parastichopus californicus	Red sea cucumber	536,275,890	498,974,195	531,192,070	452,381,885	-7.0	-14.8
Sebastes pinniger	Canary rockfish	266,563,672	256,286,022	265,664,180	226,187,759	-3.9	-14.9
Oncorhynchus kisutch	Coho salmon	117,601,463	114,363,470	117,020,635	99,367,443	-2.8	-15.1
Mytilus trossulus	Pacific blue mussel	130,607,621	125,039,446	130,308,951	110,360,681	-4.3	-15.3
Sebastes babcocki	Redbanded rockfish	198,715,465	193,186,312	195,840,938	165,753,368	-2.8	-15.4
Mesocentrotus franciscanus	Red sea urchin	128,173,401	122,413,403	125,685,423	105,872,089	-4.5	-15.8
Chionoecetes bairdi	Tanner crab	68,556,966	60,457,805	68,785,043	57,438,615	-11.8	-16.5
Microgadus proximus	Pacific tomcod	317,971,606	265,913,749	317,357,601	264,751,267	-16.4	-16.6
Oncorhynchus clarkii clarkii	Cutthroat trout	1,415,718,641	1,365,354,071	1,398,783,942	1,162,332,608	-3.6	-16.9
Sebastes maliger	Quillback rockfish	751,690,911	731,357,627	748,927,613	616,234,766	-2.7	-17.7
Sebastes borealis	Shortraker rockfish	111,056,696	108,387,931	108,488,176	89,209,471	-2.4	-17.8
Ostrea lurida	Olympia oyster	1,593,712,597	1,494,499,244	1,124,982,105	922,461,894	-6.2	-18.0
Squalus suckleyi	Spiny dogfish	92,241,419	88,223,249	91,959,365	75,362,880	-4.4	-18.0
Crassostrea gigas	Pacific cupped oyster	21,056,276	17,092,041	21,228,077	17,294,474	-18.8	-18.5
Katharina tunicata	Black chiton	422,737,947	387,047,465	420,738,668	335,287,133	-8.4	-20.3
Patinopecten caurinus	Weathervane scallop	162,631,374	153,211,096	159,111,727	125,453,656	-5.8	-21.2
Hemigrapsus oregonensis	Green shore crab	507,137,810	462,379,268	507,590,378	396,400,057	-8.8	-21.9
Pandalus borealis	Northern shrimp	44,596,402	41,187,829	44,817,849	34,913,912	-7.6	-22.1
Thunnus alalunga	Albacore tuna	1,127,888	949,419	33,614,144	25,358,745	-15.8	-24.6
Hemigrapsus nudus	Purple shore crab	288,674,187	254,616,695	290,068,685	215,153,935	-11.8	-25.8
Limanda aspera	Yellowfin sole	133,858,968	130,841,506	134,346,662	99,467,662	-2.3	-26.0
Hippoglossoides elassodon	Flathead sole	96,201,366	76,025,032	96,417,768	71,226,208	-21.0	-26.1
Sebastolobus altivelis	Longspine thornyhead	57,608,234	53,965,512	60,233,490	43,933,473	-6.3	-27.1
Eopsetta jordani	Petrale sole	90,417,852	85,121,026	88,895,350	64,633,609	-5.9	-27.3
Parophrys vetula	English sole	378,177,986	276,213,628	376,656,493	269,664,133	-27.0	-28.4
Lepidopsetta bilineata	Rock sole	88,997,015	82,934,538	87,875,592	62,931,947	-6.8	-28.4
Salvelinus malma malma	Dolly Varden trout	141,958,270	134,523,972	140,994,325	100,906,084	-5.2	-28.4

**Species**	**Common name**	**RCP 2.6**		**RCP 8.5**		**Change in catch potential (%)**	
		**2000**	**2050**	**2000**	**2050**	**RCP 2.6**	**RCP 8.5**
Citharichthys sordidus	Pacific sanddab	93,374,903	88,772,734	94,012,276	66,652,695	-4.9	-29.1
Platichthys stellatus	Starry flounder	78,698,753	74,496,781	77,863,603	55,081,614	-5.3	-29.3
Sebastes proriger	Redstripe rockfish	133,470,531	128,159,659	135,132,404	94,886,298	-4.0	-29.8
Microstomus pacificus	Pacific dover sole	54,986,545	51,705,520	55,361,304	37,829,083	-6.0	-31.7
Atheresthes stomias	Arrowtooth flounder	397,625,969	280,899,774	399,913,432	272,134,596	-29.4	-32.0
Modiolus modiolus	Northern horse mussel	43,105,992	40,837,387	43,329,165	28,698,093	-5.3	-33.8
Hemilepidotus hemilepidotus	Red Irish lord	116,552,847	111,150,124	116,260,003	76,053,662	-4.6	-34.6
Gadus macrocephalus	Pacific cod	73,649,163	68,696,170	74,449,419	48,107,193	-6.7	-35.4
Strongylocentrotus droebachiensis	Green sea urchin	46,855,839	41,415,823	46,958,697	29,922,923	-11.6	-36.3
Oncorhynchus nerka	Sockeye salmon	77,862,776	74,407,747	79,360,628	50,262,898	-4.4	-36.7
Thaleichthys pacificus	Eulachon	247,499,777	191,420,304	251,404,097	158,221,050	-22.7	-37.1
Cryptochiton stelleri	Giant Pacific chiton	50,384,441	43,571,178	50,517,805	31,672,239	-13.5	-37.3
Pandalus goniurus	Humpy shrimp	90,519,009	78,844,506	91,370,216	57,029,776	-12.9	-37.6
Glyptocephalus zachirus	Rex sole	77,818,845	50,106,069	76,558,918	46,246,023	-35.6	-39.6
Sebastolobus alascanus	Shortspine thornyhead	69,242,203	41,453,915	69,183,528	40,827,924	-40.1	-41.0
Oncorhynchus gorbuscha	Pink salmon	75,470,114	49,636,641	75,707,968	43,105,564	-34.2	-43.1
Oncorhynchus tshawytscha	Chinook salmon	118,005,595	70,114,470	118,373,616	65,955,588	-40.6	-44.3
Clupea pallasii pallasii	Pacific herring	209,013,995	150,366,200	222,629,744	113,005,876	-28.1	-49.2
Pandalopsis dispar	Sidestriped shrimp	85,519,597	49,622,096	86,428,129	24,973,631	-42.0	-71.1
Pandalus hypsinotus	Humpback shrimp	2,849,759	788,993	2,536,502	428,518	-72.3	-83.1
		**42,157,661,056**	**40,266,298,841**	**41,502,840,580**	**37,054,010,848**	**-4.5**	**-10.7**
