# Supplementary material for: Projected Scenarios for Coastal First Nations’ Fisheries Catch Potential under Climate Change: Management Challenges and Opportunities
Source: PLoS One. 2016 Jan 13;11(1):e0145285. doi: 10.1371/journal.pone.0145285 (PMC4711888; doi:10.1371/journal.pone.0145285)
Supplement: S7 Table — (PDF) [file pone.0145285.s010.pdf]

S7 Table. Sample of First Nations' traditional fisheries management approaches and analogous Western fisheries management strategies.

TRADITIONAL STRATEGY/ TOOL	DESCRIPTION	SIMILAR "WESTERN" METHODS	REFERENCE(S)
Selective fishing	Use of selective fishing gear (e.g., stone traps) or harvested species only when at a certain size (e.g., shellfish). Fished stronger runs and at specific times of year to accommodate fluctuations.	Targeted fisheries and size-selective gear	[1-3]
Goal-oriented harvesting	Pre-estimate the amount of the resource required to fulfill nutritional and economic needs, aiming to meet – rather than exceed – this quantity.	Quota-based harvests	[2]
Spatial management, or 'area licensing'	Established a system of marine space that delineated harvest zones with restricted access and limited total yields.	Management areas and limited entry	[2,4,5]
Clam gardens	Enhancement of clam beds through engineered intertidal terraces that stabilize sediments at a specific tidal height in order to increase shellfish productivity.	Mariculture	[6,7]
Monitoring	Monitored and controlled specific resources through designated individuals within a territory.	Monitoring	[8,9]
Seasonal migrations and rotational harvests	Migrated to harvesting sites to accommodate seasonal cycles in resource abundance.	Rotational or seasonal closures	[10-12]
Harvest moratorium	Closed fisheries to harvesting when stocks were weak, or prohibiting fishing for two weeks until the strength of the run was assessed (e.g., The 'First salmon rite').	Harvest closure	[13]
Adaptive ecosystem-based management	Dynamically adapt ecosystem-based management practices to test responses and modify based on feedback (e.g., clam gardens).	Adaptive ecosystem management [14]	[1,15-18]

References

1. Haggan N, Turner NJ, Carpenter J, Jones JT, Mackie Q, et al. (2004) 12,000 years of change: linking traditional and modern ecosystem science in the Pacific Northwest Victoria, BC. 16 pp. Available: http://www.fisheries.ubc.ca/webfm_send/85.

2. Menzies CR, Butler CF (2007) Returning to Selective Fishing through Indigenous Fisheries Knowledge: the example of K'moda, Gitxaala territory. American Indian Quarterly 31: 441–464.

3. White E (2011) Heiltsuk stone traps on the central coast of British Columbia. In ML Moss and A Cannon, eds. Archaeology of North Pacific Fisheries. Fairbanks, AK: University of Alaska Press. pp. 75–90.

4. Haggan N, Brown P (2003). Aboriginal fisheries issues: The west coast of Canada as a case study. In D Pauly & MLD Palomares, Production Systems in Fishery Management. pp. 17–20.

5. Powell M (2012) Divided waters: Heiltsuk spatial management of herring fisheries and the politics of Native sovereignty. Western Historical Quarterly 43(4): 463-484. Available: http://www.jstor.org/stable/westhistquar.43.4.0463.

6. Augustine S, Dearden P (2014) Changing paradigms in marine and coastal conservation: A case study of clam gardens in the Southern Gulf Islands, Canada. The Canadian Geographer / Le Géographe canadien 58: 305–314. doi:10.1111/cag.12084.

7. Groesbeck AS, Rowell K, Lepofsky D, Salomon AK (2014) Ancient clam gardens increased shellfish production: adaptive strategies from the past can inform food security today. PLOS ONE 9: e91235. doi:10.1371/journal.pone.0091235.s007.

8. Newell D (1993) Tangled webs of history: Indians and the law in Canada's Pacific Coast fisheries. Toronto, Buffalo, London: University of Toronto Press.

9. Turner NJ, Jones JT (2000) Occupying the land: Traditional patterns of land and resource ownership among First Peoples of British Columbia. pp. 1–30. Available: http://hdl.handle.net/10535/1952.

10. Turner NJ, Davidson-Hunt IJ, O'Flaherty M (2003) Living on the edge: ecological and cultural edges as sources of diversity for social-ecological resilience. Hum Ecol 31: 439–461. doi: 10.1023/A:1025023906459.

11. Turner NJ, Turner KL (2007) Traditional food systems, erosion and renewal in Northwestern North America. Indian Journal of Traditional Knowledge 6: 57–68.

12. Berkes F (2009) Indigenous ways of knowing and the study of environmental change. Journal of the Royal Society of New Zealand 39: 151–156. doi:10.1080/03014220909510568.

13. Johnsen DB (2009) Salmon, Science, and Reciprocity on the Northwest Coast. Ecology and Society 14(2): 43. Available: http://www.ecologyandsociety.org/vol14/iss2/art43/

14. Walters CJ (2007) Is adaptive management helping to solve fisheries problems? AMBIO: A Journal of the Human Environment 36: 304–307. doi:10.1579/0044-7447(2007)36%5B304:IAMHTS%5D2.0.CO;2.

15. Trosper RL (2003) Resilience in pre-contact Pacific Northwest social ecological systems. Conservation Ecology 7(3): 6. Available: http://www.ecologyandsociety.org/vol7/iss3/art6/

16. Berkes F, Colding J, Folke C (2000) Rediscovery of traditional ecological knowledge as adaptive management. Ecological Applications 10: 1251–1262.

17. Lepofsky D, Lertzman K, Hallett D, Mathewes R (2005) Climate change and culture change on the southern coast of British Columbia 2400-1200 Cal. B.P.: An hypothesis. American Antiquity 70: 267–293.

18. Council FNL (2006) BC First Nations Fisheries Action Plan. British Columbia Assembly of First Nations, First Nations Summit, and Union of British Columbia Indian Chiefs. 36 pp.
